# A distinct immune landscape in anti-synthetase syndrome profiled by a single-cell genomic study

**DOI:** 10.3389/fimmu.2024.1436114

**Published:** 2024-10-24

**Authors:** Jiayu Ding, Yanmei Li, Zhiqin Wang, Feng Han, Ming Chen, Jun Du, Tong Yang, Mei Zhang, Yingai Wang, Jing Xu, Gaoya Wang, Yong Xu, Xiuhua Wu, Jian Hao, Xinlei Liu, Guangxin Zhang, Na Zhang, Wenwen Sun, Zhigang Cai, Wei Wei

**Affiliations:** ^1^ The Province and Ministry Co-sponsored Collaborative Innovation Center for Medical Epigenetics, Department of Pharmacology, School of Basic Medical Science, Tianjin Medical University, Tianjin, China; ^2^ National Key Laboratory of Experimental Hematology, Tianjin, China; ^3^ Tianjin Key Laboratory of Inflammatory Biology, Tianjin, China; ^4^ Department of Hematology, Tianjin Medical University General Hospital, Tianjin, China; ^5^ Department of Rheumatology and Immunology, Tianjin Medical University General Hospital, Tianjin, China; ^6^ Tianjin Clinical Research Center for Rheumatic and Immune Diseases, Tianjin Science and Technology Bureau, Tianjin, China; ^7^ Department of Neurology, Tianjin Medical University General Hospital, Tianjin, China; ^8^ Department of Research and Development, Seekgene Biotechnology Co, Ltd, Beijing, China

**Keywords:** anti-synthetase syndrome (ASS), single-cell RNA sequencing (scRNA-seq), mucosal-associated invariant T (MAIT) cell, IFN-II, auto-immune diseases

## Abstract

**Objectives:**

The objective of this study was to profile the transcriptional profiles of peripheral blood mononuclear cells (PBMCs) and their immune repertoires affected by anti-synthetase syndrome (ASS) at the single-cell level.

**Methods:**

We performed single-cell RNA sequencing (scRNA-seq) analysis of PBMCs and bulk RNA sequencing for patients with ASS (N=3) and patients with anti-melanoma differentiation-associated gene 5-positive dermatomyositis (MDA5^+^ DM, N=3) along with healthy controls (HCs, N=4). As ASS and MDA5^+^ DM have similar organ involvements, MDA5^+^ DM was used as a disease control. The immune repertoire was constructed by reusing the same scRNA-seq datasets. Importantly, flow cytometry was performed to verify the results from the scRNA-seq analysis.

**Results:**

After meticulous annotation of PBMCs, we noticed a significant decrease in the proportion of mucosal-associated invariant T (MAIT) cells in ASS patients compared to HCs, while there was a notable increase in the proportion of proliferative NKT cells. Compared with MDA5^+^ DM patients, in their PBMCs ASS patients presented substantial enrichment of interferon pathways, which were primarily mediated by IFN-II, and displayed a weak immune response. Furthermore, ASS patients exhibited more pronounced metabolic abnormalities, which may in turn affect oxidative phosphorylation pathways. Monocytes from ASS patients appear to play a crucial role as receptive signaling cells for the TNF pathway. Immunophenotyping analysis of PBMCs from ASS patients revealed an increasing trend for the clone type CQQSYSTPWTF.

**Conclusion:**

Using single-cell genomic datasets of ASS PBMCs, we revealed a distinctive profile in the immune system of individuals with ASS, compared to that with MDA5^+^ DM or healthy controls.

## Highlights

- A more robust storm-like immune response was detected in PBMCs of MDA5^+^ DM patients compared to that in ASS patients.
- The frequency of MAIT cells was significantly reduced in ASS patients compared to that in healthy controls, which was verified by flow cytometry, indicating that MAIT cells may play an important regulatory role in homeostasis.
- We revealed metabolic abnormalities in ASS patients, which are involved in regulating oxidative phosphorylation pathways.
- Aberrant IFN signaling, especially the signaling mediated by IFN-II subgroup, was found in ASS.
- The ASS samples were dominated by a shared clone type CQQSYSTPWTF in their immune repertoire.

## Introduction

Idiopathic inflammatory myopathy (IIM), also known as myositis, encompasses a diverse range of autoimmune disorders with varying clinical presentations, treatment responses, and prognoses (1). Since the discovery of myositis-specific antibodies (MSAs) in 2005 and their widespread clinical application, our understanding of the subtypes associated with patients with IIMs has significantly improved (2). Anti-synthetase syndrome (ASS) is an infrequent idiopathic inflammatory myopathy characterized by the presence of at least one of three primary symptoms: myositis, interstitial lung disease (ILD), and arthritis (3). These symptoms may be accompanied by concomitant manifestations, such as mechanic’s hands and feet, Raynaud’s disease, and fever; all of these symptoms can be accompanied by the detection of synthetase antibodies in peripheral blood containing aminoacyl-tRNA. In addition to myositis, ILD represents a common and significant organ involved that contributes substantially to morbidity and mortality. Among these antibodies, Jo-1 is the most prevalent. The incidence and severity of primary and concomitant symptoms vary among these antibodies (4). Anti-melanoma differentiation-associated gene 5-positive dermatomyositis (MDA5^+^ DM) is a rare form of idiopathic inflammatory myositis that is associated with rapidly progressive, refractory ILD (5). These two diseases of ASS and MDA5^+^ DM have similar organ involvement such as the skin, muscle and lung (6, 7). And ASS presents a slower disease progression in ILD than MDA5^+^ DM (8). However, the underlying immune pathogenesis of this disease has not been fully elucidated.

In the past decade, single-cell RNA sequencing (scRNA-seq) has emerged as a promising and robust technique for analyzing gene expression in thousands of individual cells, offering an efficient and innovative tool to investigate the immune system in human diseases. It has been employed to explore uncharacterized or rare cell types or states within tissues and to elucidate dynamic changes in gene expression during cellular differentiation, temporal progression, or transitions between different cellular states (9, 10). Zhu et al. utilized scRNA-seq to provide a high-resolution depiction of the cellular landscape in peripheral blood mononuclear cells (PBMCs) derived from patients with ASS-ILD. These findings revealed an increase in interferon responses in NK cells, monocytes, T cells, and B cells among these patients. Moreover, there was a significantly greater ratio of effector memory CD8^+^ T cells to naïve CD8^+^ T cells in ASS-ILD patients than in HCs. Additionally, enrichment analysis highlighted Th1, Th2, and Th17 cell differentiation signaling pathways within the T-cell population. However, despite these insights, a comprehensive understanding of the characteristics of all PBMC types and the intricate interactions between immune cells in ASS-ILD patients has not been achieved. Furthermore, any insightful conclusions from scRNA-seq should be experimentally verified by flow cytometry or other orthogonal techniques.

In this study, we employed single-cell RNA sequencing (scRNA-seq) and bulk RNA sequencing (RNA-seq) to elucidate the cellular composition and gene expression characteristics of peripheral blood immune cells from patients with ASS. Additionally, we utilized MDA5^+^ DM as a disease control to gain more informative insights into the molecular mechanisms underlying this autoimmune disorder.

## Materials and methods

### Patients

Patients who fulfilled Solomon’s criteria for ASS (11) and Bohan and Peter’s criteria for dermatomyositis (12) at Tianjin Medical University General Hospital from July 2021 to April 2023 were recruited for the study. All patients enrolled in the study were treatment naïve. Peripheral blood samples were collected from 12 patients with ASS, 3 patients with MDA5^+^ DM and 18 healthy controls (HCs). The clinical characteristics of the patients are described in **Supplementary Tables 1**, **2**. The HCs who fulfilled the following inclusion criteria were included: (1) had normal blood test results, including blood count and liver and kidney function test results, and (2) had no symptoms of autoimmune disease. The study agreed with the recommendations of the Declaration of Helsinki. The study was approved by the hospital ethics committee of Tianjin Medical University General Hospital (approval number: IRB2021-YX-211-01), and informed written consent was obtained from the participants.

### Sample preparation

PBMCs were isolated via density gradient centrifugation with Ficoll-Hypaque (GE Healthcare, USA). The PBMCs of 3 patients with ASS, 3 patients with MDA5^+^ DM and 4 HCs were subjected to scRNA-seq, while the PBMCs of 12 patients with ASS and 14 HCs were subjected to flow cytometry.

### Single-cell RNA sequencing

**Figure 1:**
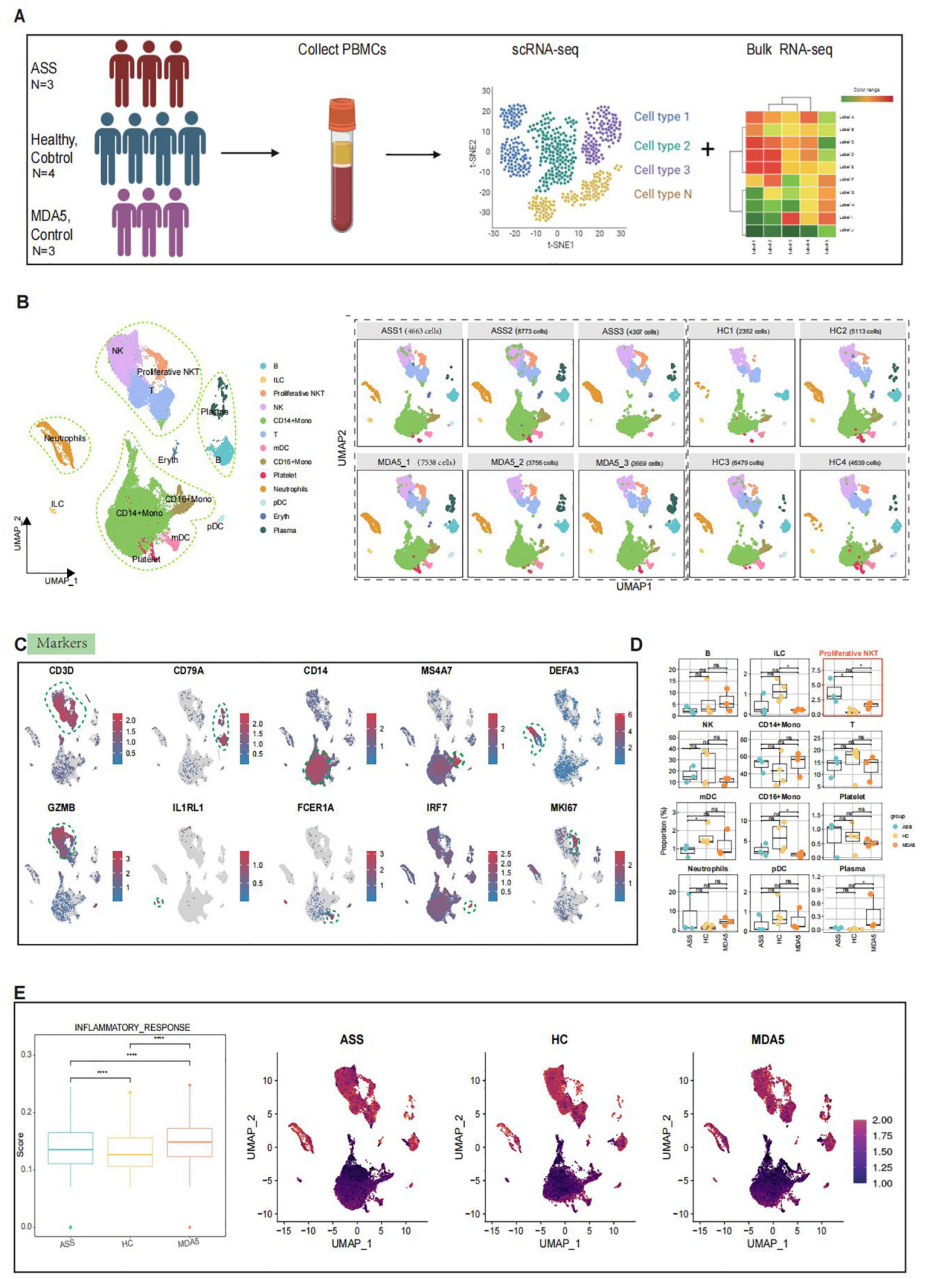
Single-cell RNA sequencing (scRNA-seq) analysis of PBMCs from ASS, HCs and MDA5^+^ DM patients. Number of samples: ASS (N = 3), HC (N=4), and MDA5^+^ DM (N=3). **(A)** Overview of the experimental workflow. **(B)** Uniform manifold approximation and projection (UMAP) plot of 13 cell types for 51, 289 high-quality single cells among PBMCs and UMAP illustrating the differentiation between controls and ASS patients based on 10 samples. **(C)** UMAP feature highlighting distinct markers associated with different cell types. **(D)** Comparison of cell subtypes among PBMCs from ASS patients and controls. **(E)** Inflammatory response scores for ASS patients and controls, along with UMAP visualizing inflammation levels across the three groups, where brighter red indicates higher inflammation levels and darker red indicates lower inflammation levels. *p<0.05; ****p<0.0001; ns, not statistically significant; Wilcoxon rank sum test.

The PBMCs were subjected to both single-cell RNA sequencing (scRNA-seq) and bulk RNA-Seq analyses (**Figure 1A**). For scRNA-seq, the experimental procedure followed the established technique with the SeekOneMe single-cell 3’ library preparation kit (SeekGene SO01V3.1, China). Batch RNA sequencing was conducted via the TruSeqTM RNA Sample Preparation Kit from Illumina (San Diego, CA, USA). The raw gene expression matrix for each sample was aggregated via the Cell Ranger (v.6.0.0) pipeline in conjunction with the human reference version GRCh38. The merged matrices were subsequently transferred to the R statistical environment for further analysis via the Seurat software package (v.4.2.0). Cells exhibiting more than 0.1% expression and those with more than 200 detected genes were selected for subsequent analysis. To ensure data quality, cells with unique mitochondrial molecular identifiers (UMIs) constituting more than 20%, as well as those with more than 4000 or fewer than 200 genes detected, were excluded as low-quality cells. Following the removal of low-quality cells, gene expression was normalized via the Harmony transformation normalization method while correcting for batch effects through the SCT integration approach. The following cells were isolated from each donor: ASS1(4663), ASS2(8773), ASS3(4307), HC1(2352), HC2(5113), HC3(6479), HC4(4639), MDA5_1(7528), MDA5_2 (3756) and MDA5_3(3669). To reduce dataset dimensionality, principal component analysis (PCA) was performed via the RunPCA function. Finally, cell clustering was accomplished by applying Find neighbors and Find clustering functions at a resolution of 1 unit. The resulting clusters were then visualized in a two-dimensional representation.

### Identifying the marker genes of single cells

The FindAllMarkers function was used to identify marker genes. Cell clusters were identified on the basis of differentially expressed genes (DEGs) exhibiting log fold changes (logFCs) (13).

### Analysis of transcription factors

SCENIC was employed as a computational tool to simultaneously reconstruct gene regulatory networks and identify stable cell states from single-cell RNA-seq data (14). The inference of the gene regulatory network was based on coexpression analysis and DNA motif analysis, followed by the examination of network activity in individual cells to determine their cellular status (14, 15). We analyzed transcription factors (TFs) using the pySCENIC package (16).

### Cell–cell communication analysis

The signaling inputs and outputs among the different cell types and cell clusters were assessed using the CellChat package (17). The netVisual_circle function was employed to evaluate the strength of cell−cell communication networks within specific subsets of cells (17).

### Ligand−receptor analysis

NicheNet focuses on the analysis of cell-to-cell communication, considering not only potential interactions between ligands and receptors but also the incorporation of information on signaling networks and target genes that may be impacted (18, 19). Developed by the Saeys Laboratory and made available on GitHub (https://github.com/saeyslab/nichenetr), NicheNet aims to predict how one cell (the sender cell) influences the gene expression of another cell (the receiver cell) through ligand secretion.

### Immune repertoire analysis

The Immunarch (v 1.0.0) package is specifically designed for the analysis of T-cell receptors (TCRs) and B-cell receptors (BCRs). (Immunarch: an R package for painless bioinformatics analysis of T-cell and B-cell immune repertoires. Zenodo https://doi.org/10.5281/zenodo.3367200 (2019))

### ImmuCellAI analysis

ImmuCellAI is an immune cell abundance assessment method based on a gene set signature. It is a tool for accurately estimating the abundance of 24 immune cell types (18 T-cell subsets) from gene expression data (20).

### scMetabolism analysis

scMetabolism is an R package used to quantify metabolism at the single-cell level (21). The package is based on a conventional single-cell matrix file, to score each cell, and uses the algorithm of VISION cells to obtain each activity score of metabolic pathways at the single-cell level to evaluate the characteristics of cell metabolism. Analyses were performed with the use of the Seurat software package (v.4.2.0).

### TRUST4 analysis

TRUST4 is an algorithm that can identify immune cell receptor sequences from bulk RNA-seq and single-cell RNA-seq data (22). (Website: https://github.com/liulab-dfci/TRUST4)

### Pseudotime analysis

The Vector package was used to perform the pseudotime analysis. The Vector packages aims to infer the vector of cell development and bases on the grid distance of the starting cells in UMAP. The cell principal component value quantile polarization is closely related to the development level in the state to determine the primitive cells (23).

### Flow cytometry

PBMCs were first stained with a Zombie NIR™ Fixable Viability Kit (BioLegend) to remove dead cells. PerCP-conjugated anti-human CD3 (BioLegend) and the 5-OP-RU MR1 tetramer were used for surface staining in the present study. Flow cytometry analysis was performed with FlowJo software (BD).

### Statistical analysis

Statistical analyses were conducted using Prism 9 or R. Differences in quantitative parameters were evaluated using the t test for normally distributed data or nonparametric tests for data that were not normally distributed. Unless otherwise specified, the results with a *P* value < 0.05 were considered statistically significant. * *P <*0.05; ** *P <*0.01; *** *P <*0.001; **** *P <*0.0001; ns, not statistically significant.

### Data availability

The raw sequencing data from this study have been deposited in the Genome Sequence Archive in BIG Data Center (https://bigd.big.ac.cn/), Beijing Institute of Genomics (BIG), Chinese Academy of Sciences, under the accession number: PRJCA024528. Any additional information required to reanalyze the data reported in this paper is available from the lead contact upon request.

## Results

### Single-cell sequencing of human peripheral blood revealed the presence of distinct cellular subpopulations

The clinical characteristics of each patient are presented in **Supplementary Table 1**. Following quality control measures, a total of 51,289 PBMCs were utilized for further analysis, comprising 17,743 cells from three ASS patients, 18,538 cells from four controls, and 14,963 cells from three MDA5^+^ DM patients. On the basis of classical gene expression markers (24), the cells were categorized into 13 subgroups: T cells (*CD3D*), B cells (*CD79A*), CD14^+^ monocytes (*CD14*), plasma cells (*JCHAIN*), CD16^+^ monocytes (*MS4A7*), neutrophils (*DEFA3*), natural killer cells (*GZMB*), innate lymphocytes (*IL1RL1*), myeloid dendritic cells (*FCER1A*), plasmacytoid dendritic cells (*IRF7*), platelets (PPBP), erythrocytes (*HBB*) and proliferative NKT cells (*MKI67*, *CD3D*, *GZMB)* (**Figures 1B, C**; **Supplementary Figure 1A**). Interestingly, multiple cell clusters with elevated expression of the interferon-related gene *IFITM2*, inflammation-related genes (*IER2*, *S100A8*, and *FOS*) and cytoskeleton-related genes (*ACTB*) were observed in ASS patients compared to healthy controls (**Supplementary Figures 1D, E**). Multiple clusters of cells with heightened expression of proinflammatory cytokines (*IL1B*) and HLA class II-related genes (*HLA_DRB5*) were found in ASS patients compared to MDA5^+^ DM controls. Additionally, MDA5^+^ DM patients displayed more than one cell cluster with high expression of interferon genes (*IFIT1*, *IFIT2*, *IFIT3*, *IFI44*, *IRF7*, *IFI127*, *IFIH1*, *IFI27 and IF16*), as well as interferon-stimulated genes (*MX1* and *MX2*). This suggests that the immune response in both diseases is associated with the interferon pathway. Second, the proportion of proliferative NKT cells in patients with ASS was greater than that in both the HC group (*P*=0.045) and the MDA5^+^ DM group (*P*=0.099), indicating a potential association between proliferative NKT cells and the progression of ASS disease (**Figure 1D**).

Then, utilizing scMetabolism (21), which is used for quantifying single-cell metabolism levels, we observed abnormally enhanced metabolic activities in ASS patients, particularly in the tricarboxylic acid cycle (TCA cycle) (**Supplementary Figure 1B**). To investigate alterations in different immune response signaling pathways among the three groups, we employed the signature gene set from the Molecular Signature database (MsigDB) to score inflammatory responses. The greatest inflammatory response was observed in MDA5^+^ DM patients, followed by ASS patients, with increased enrichment scores observed for CD14^+^ Mono subset T cells, neutrophil subsets, and proliferative NKT subsets (**Figure 1E**). Furthermore, enrichment of genes in the Gene Ontology (GO) gene set for antigen presentation, type I interferon signaling pathway, type II interferon pathway and oxidative phosphorylation pathway across all three groups revealed that immune response-related pathways were not as strongly activated in the PBMCs of ASS patients compared to those in the PBMCs of MDA5^+^ DM patients (**Supplementary Figure 1C**). These results are consistent with the clinical features of ASS and MDA5^+^ DM.

### T cells present in PBMCs

**Figure 2:**
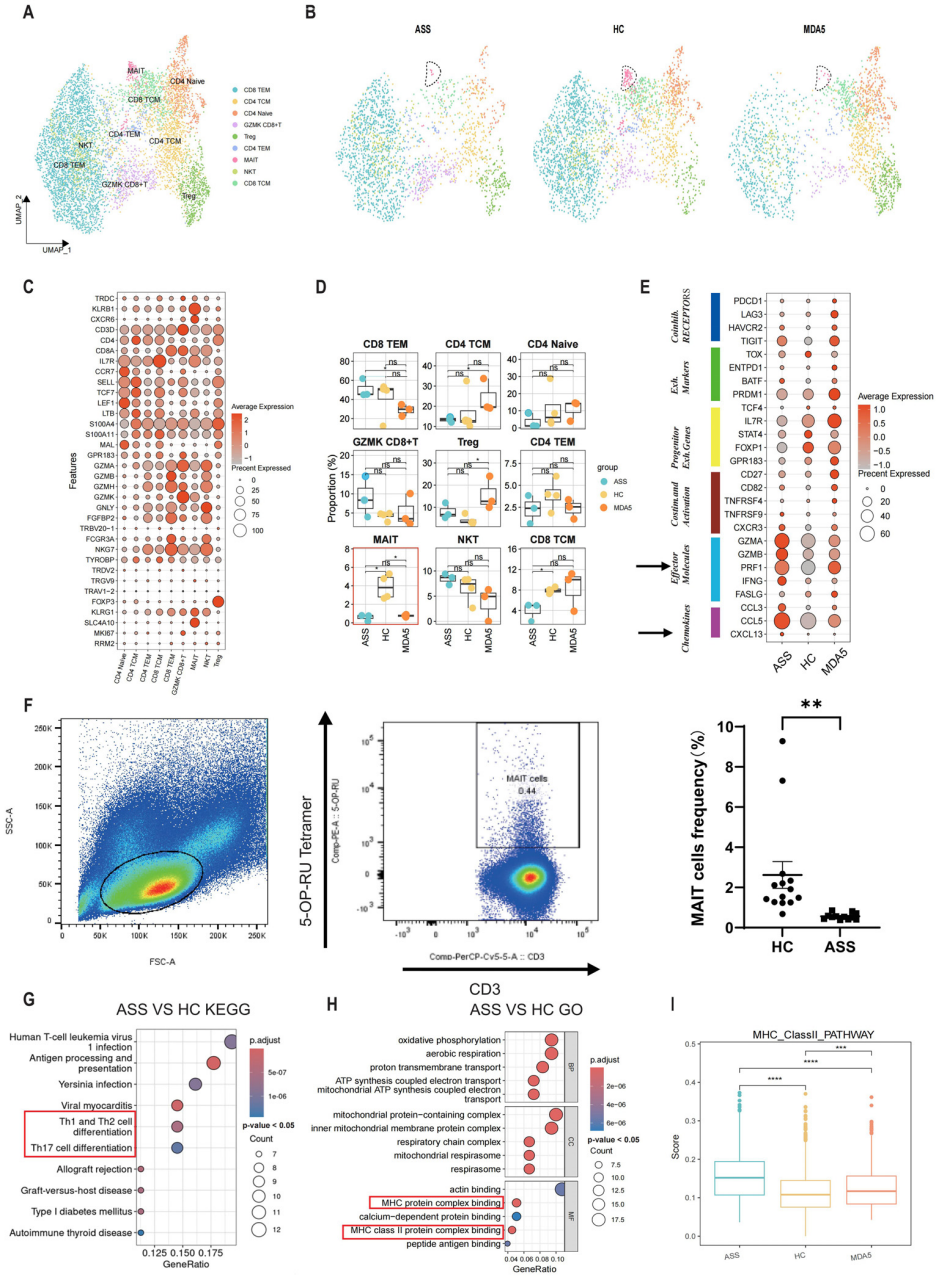
The transcriptional profile of PBMCs was compared between patients with ASS and control subjects to assess changes in T-cell at a single-cell level. Number of samples: ASS (N = 3), HC (N=4), MDA5^+^ DM (N=3). **(A)** Unified manifold approximation and projection (UMAP) plot showing the nine T-cell subtypes from ASS patients and controls. **(B)** UMAP plot of T cells in the control and ASS patient groups. **(C)** Bubble plots illustrating the markers of different T-cell types. **(D)** Boxplot of T-cell subtypes in PBMCs from ASS patients and controls. *p<0.05; ns, not statistically significant; Wilcoxon rank sum test. **(E)** T-cell function in ASS patients and controls. **(F)** Representative flow cytometry analysis and statistics for MAIT cells in the ASS patient and HC groups. **(G)** KEGG enrichment analysis of genes upregulated in T cells between ASS patients and HCs. The top 10 pathways which had a p < 0.05 were presented. **(H)** GO enrichment analysis of upregulated genes in T cells comparing ASS patients with HCs. The top 10 pathways which had a p < 0.05 were presented. **(I)** MHC type II pathway scores of the three groups; each dot represents the score of a single cell; *p<0.05; **p<0.01; ***p<0.001; ****p<0.0001; Wilcoxon rank sum test.

Due to the crucial role of T cells in the progression of ASS disease, we subsequently isolated and reaggregated T cells from the entire pool (**Supplementary Figure 2A**). By analyzing the expression levels of *TDDC*, *KLRB1*, *CXCR6*, *CD3D*, *CD4*, *CD8A*, *IL7R*, *CCR7*, *SELL*, *TCF7*, *LEF1*, *LTB*, *S100A4*, *S1LLA11*, *MAL*, *GPR183*, *GZMA*, *GZMB*, *GZMH*, *GZMK*, *GNLY*, *FGFBP2*, *TRBV20-1*, *FCGR3A*, *NKG7*, *TYROBP*, *TRDV2*, *TRGV9*, *TRAV1-2*, *FOXP3*, *KLRG1*, *SLC4A10*, *MKI67* and *RRM2* (**Figure 2C**), we identified nine distinct types of T cells, including effector memory CD8^+^ T lymphocytes (CD8 TEM), effector memory CD4^+^ T lymphocytes (CD4 TEM), naïve CD4^+^ T lymphocytes (CD4 naïve), GZMK-expressing cytotoxic CD8^+^ T cells (GZMK CD8^+^ T), regulatory CD4^+^ T lymphocytes (Treg), central memory CD4^+^ T lymphocytes (CD4 TCM), central memory cytotoxic CD8^+^ T cells (CD8 TCM), NKT cells and mucosal-associated invariant T (MAIT) cells (**Figures 2A, B**; **Supplementary Figure 2B**). The proportion of MAIT cells was lower in ASS patients than in HCs (**Figure 2D**). Compared to those of HCs, ASS patient-derived MAIT cells exhibited greater expression of inflammation-related genes such as *IFNG*, *IFITM2* and *STAT1* (**Supplementary Figure 2C**). Then, flow cytometry was used to validate the alterations in the frequency of MAIT cells in 12 treatment-naïve ASS patients and 14 HCs. The proportion of MAIT cells was decreased in the ASS group (**Figure 2F**). The percentage of cells expressing marker genes related to T-cell function, including coinhibitory receptor interaction genes, exhaustion-related genes, exhaustion precursor genes, stimulation and activation genes, effector molecules, and chemotactic migration genes, was greater in the ASS group (**Figure 2E**). In addition, the results of GO (gene ontology) and KEGG (Kyoto Encyclopedia of Genes and Genomes) analyses revealed significant enrichment of “antigen processing and presentation”, “Th1 and Th2 cell differentiation”, “Th17 cell differentiation”, “MHC protein complex binding” and “MHCII protein complex binding” in T cells from ASS patients. These findings suggest that inflammation and antigen presentation play crucial roles in the pathogenesis of ASS (**Figures 2G, H**). Furthermore, the ASS group presented higher scores for the type II interferon pathway and oxidative phosphorylation pathway (**Figure 2I**; **Supplementary Figure 2D**). CellChat was used to investigate intercellular communication across multiple T-cell subtypes. The results indicated that GZMK CD8+ T cells and CD4+ naïve cells were key players involved in cell signaling communication within the ASS group (**Supplementary Figure 2E**).

### The features of proliferative NKT cells, monocytes and neutrophils in patients with ASS

**Figure 3:**
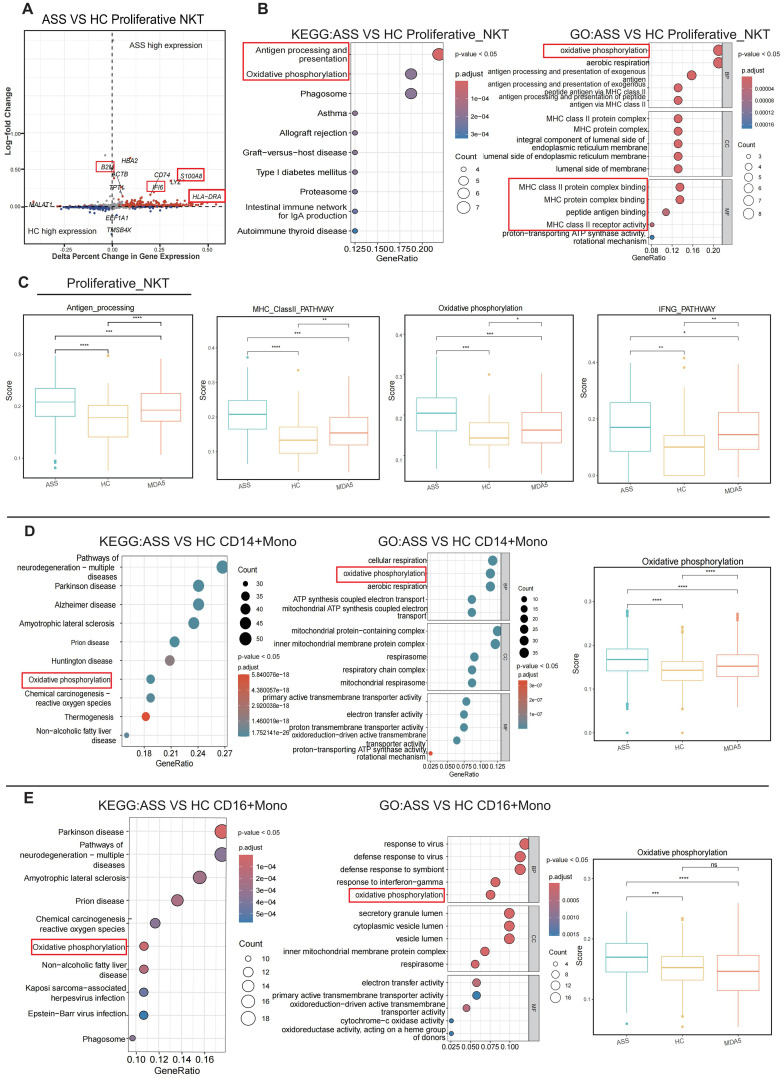
The aberrant appearance of proliferative NKT cells and monocytes in patients with ASS. Number of samples: ASS (N=3), HC (N=4), and MDA5^+^ DM (N=3). **(A)** Volcano plot showing the differentially expressed genes. The gray scatter points represent nonsignificantly differentially expressed genes, whereas the red and blue scatter points represent significantly differentially expressed genes. The red font indicates the genes we focused on. **(B)** Enrichment analysis of proliferative NKT cells. **(C)** Antigen presentation score, type II MHC pathway score, oxidative phosphorylation pathway score, and IFNG pathway score for both the ASS patient group and the control group. *p<0.05; **p<0.01; ***p<0.001; ****p<0.0001, Wilcoxon rank sum test. **(D)** KEGG enrichment analysis, GO analysis, and oxidative phosphorylation pathway score in CD14^+^ monocytes from ASS patients. ****p<0.0001; Wilcoxon rank sum test. **(E)** KEGG enrichment analysis, GO analysis, and oxidative phosphorylation pathway score in CD16^+^ monocytes from ASS patients. ***p<0.001; ****p<0.0001; ns, not statistically significant; Wilcoxon rank sum test.

Differentially expressed gene (DEG) analysis revealed high expression of *B2M*, *IL16*, *LYZ*, *S100A8*, and *HLA-DRA* as well as inflammation- and immune-related genes in ASS patients (**Figure 3A**). Due to the significant difference in the proportion of proliferative NKT cells between the patient group and the HC group, we analyzed the characteristics of proliferative NKT cells. KEGG and GO analyses revealed high enrichment of antigen processing and presentation pathways, MHC-related pathways, and oxidative phosphorylation pathways in proliferative NKT cells of ASS patients (**Figure 3B**). The ASS patient group exhibited the highest scores in terms of antigen presentation, type II interferon, and the oxidative phosphorylation pathway (**Figure 3C**). These findings suggest the significant involvement of proliferative NKT cells in ASS pathogenesis. Then, innate immune cells such as monocytes and neutrophils were re-clustered. KEGG and GO analyses of monocytes revealed that oxidative phosphorylation reaction pathways were enriched within CD14^+^ and CD16^+^ Mono cells in the ASS group (**Figures 3D, E**). In addition, the CD14^+^ monocyte cell subsets can be further divided into three subtypes: CD14_Activated (*PLBD1*, *ALOX5AP*, *TSPO*, *HP*, *S100A8*, *S100A12*, and *CTSD*), CD14_HLA (*CD74* and *HLA-DPB1*), and CD14_ISG (*MX1*, *LY6E*, *ISG15*, *XAF1*, and *FCER1A*) (**Supplementary Figures 3A, B**). The proportion of CD14_Activated cells showed the greatest change in ASS patients (**Supplementary Figure 3C**). The differential expression analysis (DEG) revealed that the *TNF* and *IFI27* genes were upregulated in the ASS patient group and that both genes were associated with the inflammatory response (**Supplementary Figure 3D**). GO analysis of the three subtypes of CD14^+^ monocytes revealed that CD14_Activated cells were significantly enriched in pathways associated with myeloid cell migration (**Supplementary Figure 3E**). Neutrophils were categorized into four types based on highly expressed genes: MKI67_Neu, CAMP_Neu, MMP9_Neu, and CTSG_Neu (**Supplementary Figures 3F, G**). The patients with ASS exhibited the most significant alteration in the proportion of CAMP_Neu cells (**Supplementary Figure 3H**). Consequently, we conducted DEG of CAMP_Neu cells within the ASS group and revealed that both the *IF16* and *B2M* genes were upregulated. Notably, these genes are closely associated with the inflammatory response and immune response (**Supplementary Figure 3J**). Interestingly, trajectory analyses of neutrophils revealed that the MKI67_Neu cell type differentiated into other cell types in the patient group, while CAMP_Neu cells were the origin of development in HCs, indicating that neutrophils may be involved in the development of ASS (**Supplementary Figure 3I**).

### Analysis of intercellular communication and ligand−receptor interactions

**Figure 4:**
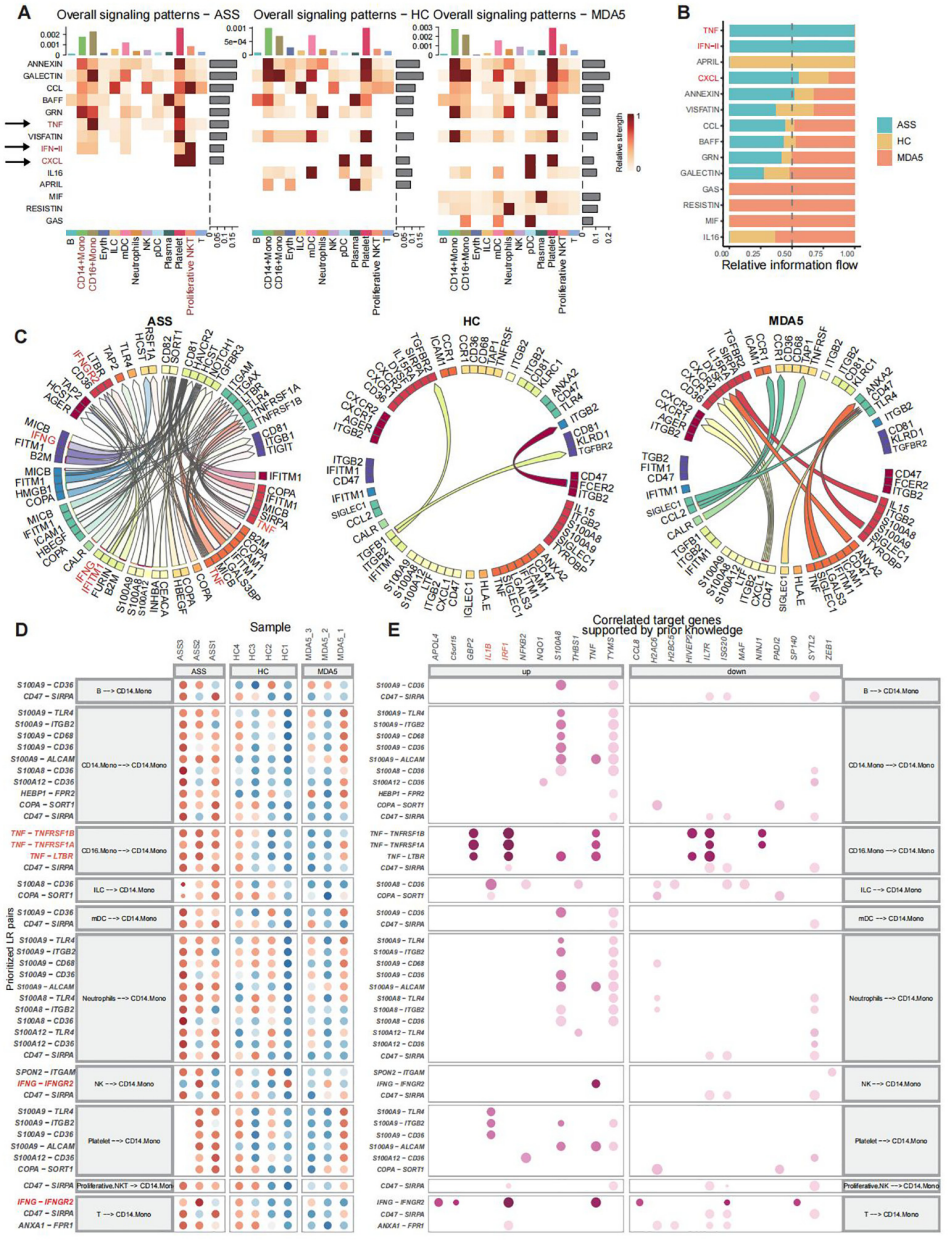
Analysis of ligand−receptor interactions and cellular communication in scRNA-seq of PBMCs from patients with ASSs and controls. Number of samples: ASS (N=3), HC (N=4), and MDA5^+^ DM (N=3). **(A)** The predominant contribution of efferent and afferent signals to the 13 cell populations, with darker shades of red indicating stronger effects. **(B)** Ranking of signaling pathways among the three groups. **(C)** Three groups of overall ligand−receptor pairs. Arrows point from ligand to receptor. **(D)** CD14^+^ Mono cells presented as a ligand−receptor pair for the receptor. **(E)** The target genes associated with ligand−receptor pairs.

CellChat was used to quantify intercellular communication across diverse cell populations. CD14^+^ Mono cells, CD16^+^ Mono cells, and proliferative NKT cells, which were activated in patients with ASS, engaged extensively in interactions with various immune cell subsets and were identified as major contributors to efferent or transferred signals (**Figure 4A**; **Supplementary Figure 4B**). The TNF signaling pathway, IFN_II signaling pathway, and CXCL signaling pathway were more enriched in the ASS patient group than those in the control group (**Figure 4B**; **Supplementary Figure 4A**). Moreover, the number of Ligand−Receptor interactions, including interactions with IGNG, B2M, and IFITM1, was significantly greater in ASS patients than in controls. TNFRSF1A, TNFRSF1B, and IFNGR2 acted as ligands in ASS group (**Figure 4C**). In addition, ligand−receptor interaction analysis revealed that the TNF−TNFRSF1A, TNF−TNGRF1B, and TNF−LTBR ligand pairs were more active in CD16^+^ Mono cells than those in CD14^+^ Mono cells in the ASS group (**Figure 4D**). In patients with ASS, ligand−receptor link target genes, including *IRF1*, *IL1B*, and *TNF*, were upregulated (**Figure 4E**; **Supplementary Figures 4C, D**).

### Transcription factor regulation analysis of various cell types

**Figure 5:**
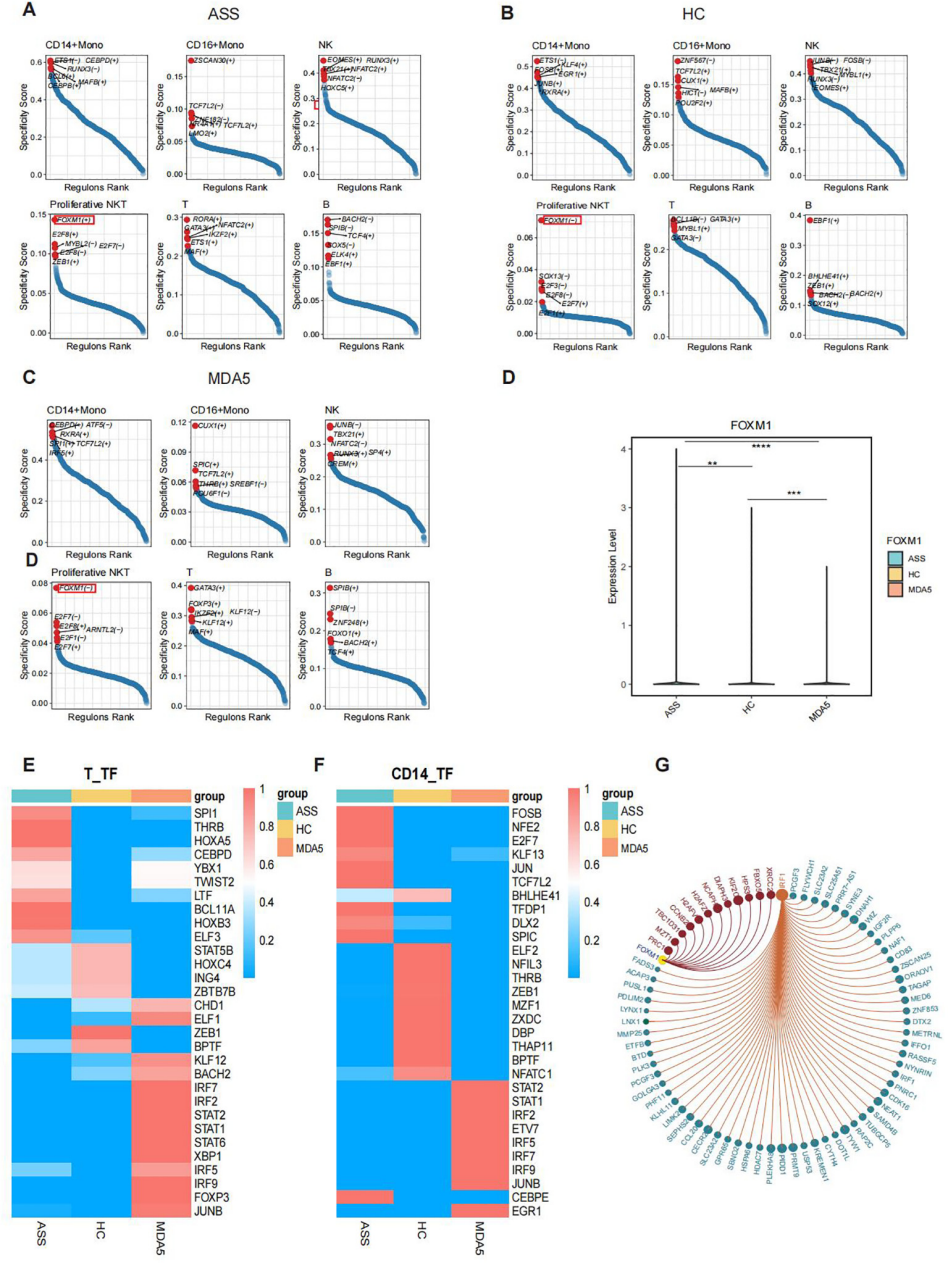
Transcription factor analysis of PBMCs from patients with ASS and controls via scRNA-seq. Number of samples: ASS (N=3), HC (N=4), and MDA5^+^ DM (N=3). **(A)** Transcription factor-encoding genes specific for different cell subsets in the ASS patient group. (+) indicates positive regulation, and (-) indicates negative regulation. **(B)** Transcription factors specific for different cell subsets in HCs. **(C)** Transcription factors specific for different cell subsets in the MDA5^+^ DM patient group. **(D)** Expression levels of the *FOXM1* gene in ASS patients and controls. **(E)** Heatmap showing the top 10 transcription factor active genes in T cells from ASS patients and controls. **(F)** Heatmap showing the top 10 transcription factor sets in CD14^+^ monocytes from ASS patients and controls. **(G)** Target genes of the transcription factors IRF1 and FOXM1. **p<0.01; ***p<0.001; ****p<0.0001, Wilcoxon rank sum test.

We conducted transcription factor specificity analyses on CD14^+^ Mono, CD16^+^ Mono, NK, proliferative NKT, T, and B cells from the three groups. Our investigation revealed that FOXM1 was specifically upregulated in ASS cells but downregulated in MDA5^+^ DM cells and HCs among proliferative NKT cells (**Figures 5A–D**). Compared with HCs, transcription factors of SPI1, THRB and HOXA5 had higher activity in T cells of ASS patients (**Figure 5E**). Additionally, the IRF1 transcription factor displayed significantly elevated activity in all three ASS patients (**Supplementary Figures 5A–D**). Notably, the FOSB, JUN, and NFE2 transcription factors exhibited increased activity specifically in CD14^+^ Mono cells within the ASS patient group (**Figure 5F**; **Supplementary Figures 5E, F**). Based on these results, we identified FOXM1 and IRF1 as potential key regulatory transcription factors involved in ASS disease for target gene prediction purposes. The target genes of FOXM1 include *PRC1* and *MZT1*, while the target genes of IRF1 include *PCGF3* and *SLC23A2* (**Figure 5G**).

### Analysis of the immune repertoire using single-cell RNA-seq and bulk RNA-seq data analysis

**Figure 6:**
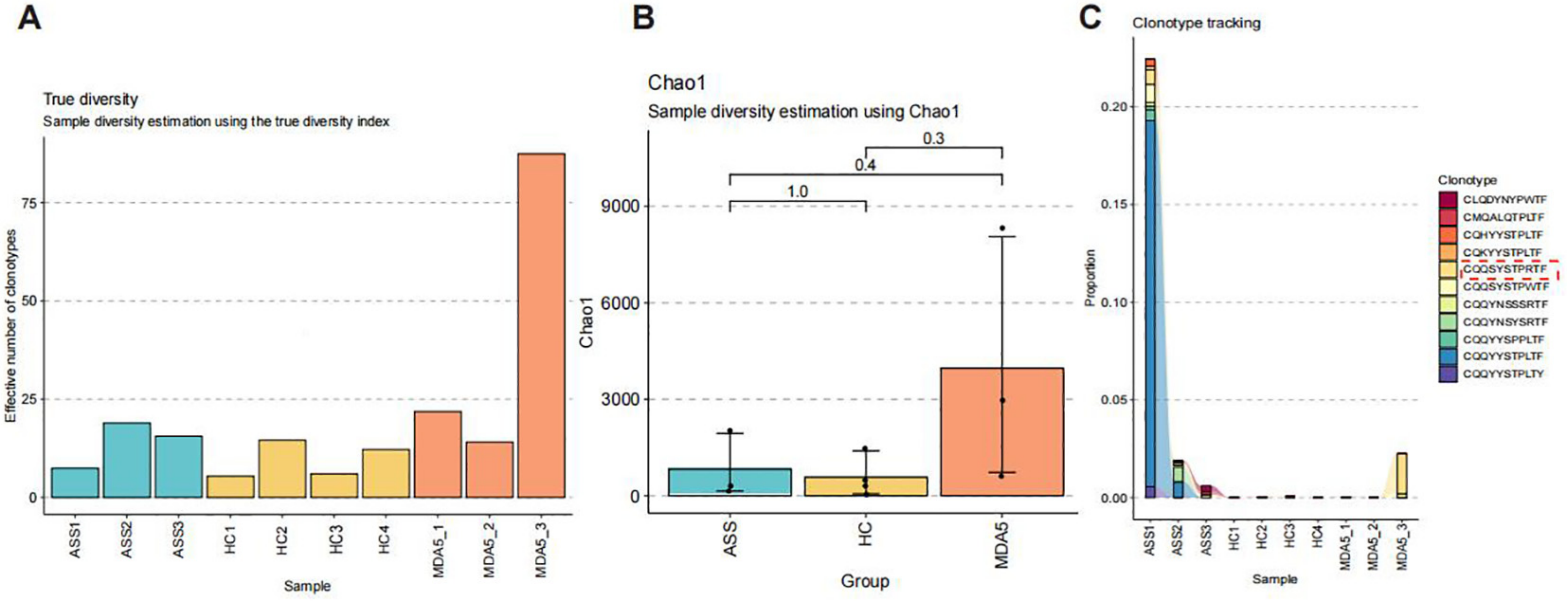
Number of samples: ASS (N=3), HC (N=4), MDA5^+^ DM (N=3). **(A)** Number of distinct clone types observed in 10 samples. **(B)** The Chao1 index in patients with ASS, HCs, and patients with MDA5^+^ DM. **(C)** Tracking the clonotypes in patients with ASS, HCs, and patients with MDA5^+^ DM.

TCR and BCR libraries were extracted from single-cell RNA-seq data by TRUST4 and analyzed using the immunome library package. The number of all clones and the number of unique clonotypes were greater in ASS patients than that in HCs (**Figure 6A**). Although not significantly different, the TCR and BCR repertoire richness estimated using Chao1 in ASS patients tended to be greater than that in HCs (**Figure 6B**). The analysis revealed that the complementarity determining region 3 (CDR3) length distribution of immune cells from ASS patients was skewed toward longer sequences and peaked at 11 amino acids (**Supplementary Figure 6A**). We traced clonotypes in patients with ASS, HCs, and MDA5^+^ DM and found that the clonotypic expansion of one CDR3 amino acid, CQQSYSTPWTF, occurred in all three ASS patients (**Figure 6C**).

### Analysis of bulk RNA-seq data analysis

**Figure 7:**
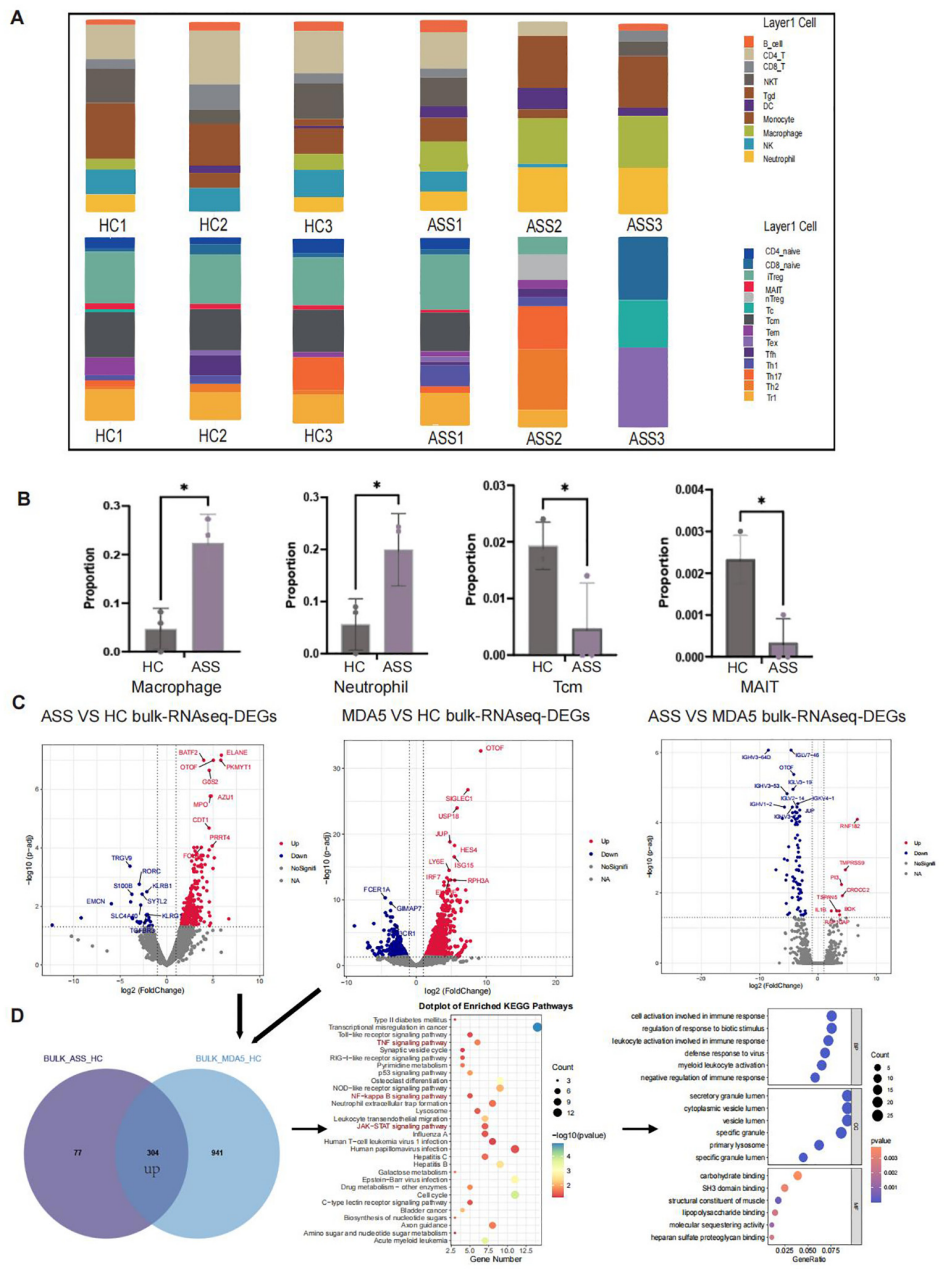
Number of samples: ASS (N=3), HC (N=3), and MDA5^+^ DM (N=3). **(A)** Bulk RNA-seq ImmuCellAI analysis of ASS, HCs and MDA5^+^ DM patients. **(B)** Statistics of the ImmuCellAI analysis results. **(C)** Comparison of DEGs in PBMC bulk RNA-seq between patients with ASS and HCs (red: up in ASS), between patients with MDA5^+^ DM and HCs (red: up in MDA5+ DM), and between ASS patients and MDA5^+^ DM patients (red: up in ASS). **(D)** Venn diagram showing upregulated genes in ASS patients and upregulated genes in MDA5^+^ DM patients according to bulk RNA-seq and KEGG and GO pathway enrichment analyses. *p<0.05.

To verify the results of single-cell sequencing, we used ImmuCellAI to estimate the infiltration abundance of 24 immune cells on the basis of gene expression datasets, including bulk RNA-Seq data (**Figure 7A**). The results revealed that the numbers of macrophages and neutrophils were significantly greater, whereas the numbers of TCM cells and MAIT cells were significantly lower in ASS patients than in HCs (**Figure 7B**). Further analysis of bulk RNA-seq data revealed that the expression of the *BATF2*, *ELANE*, and *GOS2* genes, which are associated with cell cycle regulation, was upregulated in patients with ASS compared with HCs. Additionally, the expression of the *MPO* and *ELANE* genes, which are related to neutrophil function, was also upregulated. Our findings demonstrated that the *RNF182* gene was upregulated in patients with ASS compared with patients with MDA5^+^ DM, which is consistent with our previous analysis of single-cell sequencing data (**Figure 7C**). We subsequently examined the similarities between the bulk RNA-seq data from the ASS group and the MDA5^+^ DM group. A total of 304 genes were upregulated in both the ASS vs HC and MDA5^+^ DM vs HC comparisons. KEGG analysis revealed that TNF signaling, NF-kappaB signaling, JAK-STAT signaling, and other inflammation-related pathways were upregulated (**Figure 7D**). Furthermore, the *CTSA* and *SOCS3* genes presented high expression levels in the myeloid cell population according to bulk RNA-seq data from the ASS patient group, suggesting their potential as biomarkers for ASS patients (**Supplementary Figures 6B, C**).

## Discussion

Understanding the pathogenesis of ASS is helpful for identifying biomarkers and developing targeted treatment strategies. The 3 ASS patients included in this study had rash, myositis and ILD, so we chose MDA5^+^ DM patients as disease control (25). However, these two diseases differ significantly in terms of the types of antibodies involved, disease progression and prognosis. By comparing the single-cell transcriptional landscape in PBMCs between ASS patients and those with MDA5^+^ DM, we aimed to gain further insights into the pathogenesis of both diseases.

Our findings from single-cell sequencing revealed significant differences in cell type proportions, including a decrease in MAIT cells and an increase in proliferative NKT cells within the ASS patient group. The significant reduction in the proportion of MAIT cells among ASS patients was further confirmed by both bulk RNA-seq data analysis and flow cytometry. MAIT cells represent a phylogenetically conserved subset of T cells, and in humans, the frequencies of MAIT cells in the blood are altered during various autoimmune diseases and are often associated with dysregulation of the microbiota (26). Our findings align with previous studies that reported an enrichment of Th1, Th2, and Th17 cell differentiation signaling pathways in T cells within PBMCs from individuals with ASS (10). Further investigation revealed that a population of proliferative NKT cells was highly enriched in antigen processing and presentation pathways, MHC-related pathways and oxidative phosphorylation pathways in ASS patients and had significantly greater scores in related pathways than in the MDA5^+^ DM patient group. Moreover, the FOXM1 transcription factor is highly expressed only in ASS patients and plays a positive regulatory role. However, further studies are needed to explore the roles of MAIT cells and NKT cells in ASS.

Previous reports have indicated significantly lower survival rates among MDA5^+^ DM-ILD patients than among those with ASS-ILD (27). In our study, we found that, compared with ASS patients, MDA5^+^ DM patients presented higher inflammatory scores. The routine myo-pathological diagnosis of inflammatory/immune dysregulation myopathies must include HLA-DR testing, as it has been demonstrated that ASS is characterized by robust MHC-II/HLA-DR expression in muscle fibers exhibiting a distinctive peripheral pattern. It is hypothesized that the presence of *HLA-DR* expression in ASS may indicate a specific immune mechanism potentially involving IFNγ (28). Our study revealed upregulation of the *HLA_DRB5* gene in multiple cell populations among ASS patients compared to MDA5^+^ DM patients. Although this finding was derived from single-cell analysis of peripheral blood, it implies potential circulation and associations between peripheral blood and muscle fiber tissue.

Metabolic disorders can lead to irreversible structural damage in the muscle fibers of patients with IIMs. There have been studies that identified enriched metabolic pathways, including tryptophan metabolism, phenylalanine and tyrosine metabolism, fatty acid biosynthesis, β-oxidation of very long-chain fatty acids, α-linolenic and linoleic acid metabolism, steroidogenesis, bile acid biosynthesis, purine metabolism, and caffeine metabolism, in DM and ASS (29). Notably, our results also showed that multiple metabolic pathways were abnormally activated in ASS patients. KEGG and GO analyses revealed that oxidative phosphorylation pathways were highly enriched in multiple cell types of ASS patients. And ASS patients exhibited the highest pathway score of oxidative phosphorylation pathway, followed by MDA5^+^ DM group. It has been reported that there is significant enrichment across various metabolic pathways, including oxidative phosphorylation and pyruvate metabolism, in MDA5^+^ DM patients (30). The autoreactive CD4 T-cell effector subsets (Th1 and Th17) that drive the pathogenesis of these diseases exhibit an increased reliance on glycolytic metabolism to upregulate key transcription factors, including T-bet and RORγt, which play crucial roles in their differentiation and proinflammatory responses. However, studies on immunometabolism have demonstrated that mitochondria-derived reactive oxygen species (ROS) serve as signaling molecules that contribute to the fate and function of T cells. Targeting glycolysis or ROS production to eliminate autoreactive T cells is a potential strategy for inhibiting their activation without compromising systemic immune function (31). Metabolic abnormalities are associated with an inflammatory immune cell phenotype, which has been implicated in the pathogenesis of autoimmune diseases. Therefore, investigating immune metabolism related to the oxidative phosphorylation pathway is anticipated to offer novel opportunities for monitoring and treating ASS.

Cell-to-cell communication analysis revealed significant differences in the TNF pathway, IFN-II signaling pathway, and chemokine signaling pathway among the ASS patient groups. Moreover, myeloid cells and proliferative NKT cells have emerged as the predominant contributors to efferent or transferred signals. Originally identified for its role in inducing tumor necrosis, tumor necrosis factor α (TNFα) has recently been recognized for its crucial involvement in autoimmune diseases as a pathological component. TNFα binds to two distinct receptors and initiates signal transduction pathways that elicit diverse cellular responses, including survival, differentiation, and proliferation (32). These findings suggest that TNFα may be a potential therapeutic target. The cytokine IFNγ is primarily produced by cells of the innate immune system, including monocytes, macrophages, and natural killer (NK) cells (33, 34). IFNγ is primarily produced by cells of the innate immune system, including monocytes, macrophages, and NK cells. Interestingly, in muscle biopsies from patients with ASS, CD8^+^ T cells were found to be in close proximity to MHC class II+ muscle fibers, suggesting the involvement of the IFN-II pathway (35). Thus, analysis of RNA sequencing data from a substantial number of myositis muscle biopsies revealed significant activation of IFN-II in patients with ASS, while the expression of genes induced by IFN-I was only modestly observed in ASS (36). Histidine RNA synthetase (HRS)-reactive CD4^+^ T cells, which exhibit a Th1 phenotype and produce elevated levels of IFNγ, have been detected in bronchoalveolar lavage fluid from patients with ASS (37). In the peripheral blood of ASS patients, the presence of HRS-reactive CD4^+^ T cells has also been reported (37); however, Th1 cell involvement is less pronounced in the lungs. Collectively, these data support the role of IFN in ASS. Transcriptomic studies suggest that IFN-II plays a more prominent role than IFNI (35), which aligns with the results obtained from our single-cell transcriptomic analysis of peripheral blood samples. Based on the aforementioned findings, we can conclude that disease activity is associated with IFN-induced gene expression, which exhibits distinct IFN signatures in ASS and MDA5^+^ DM patients. Targeting these pathways may offer potential therapeutic approaches for this disease. However, changes observed by CellChat in cells present in peripheral blood may reflect residual, somewhat durable gene expression changes that likely originated from interactions originally occurring in tissues.

T cells play a crucial role in the adaptive immune system, and analyzing T-cell receptors (TCRs) in the peripheral blood of individuals with ASS can offer valuable insights into disease pathogenesis. Through immune repertoire analysis, we identified an amplified CDR3 amino acid clonotype, CQQSYSTPWTF, in ASS patients, providing evidence for a shared antigen-driven T-cell response under these conditions. Furthermore, we will investigate the presence of disease-associated antigen-specific clones surrounding the disorder to potentially increase diagnostic accuracy and facilitate disease stratification.

Additionally, we integrated scRNA-seq and Bulk-seq data to identify commonalities between ASS diseases and MDA5^+^ DM diseases. Finally, we integrated the scRNA-seq and Bulk-seq data to identify commonalities that were enriched in signaling pathways associated with Th1 and Th2 cell differentiation, Th17 cell differentiation, and NOD-like receptor activation, suggesting the involvement of inflammation and antigen presentation in both diseases. Through bulk-seq data analysis, we observed the joint upregulation of 304 genes in these two diseases compared with HCs. These genes were significantly enriched in inflammation-related signaling pathways, such as the TNF signaling pathway, NF-kappaB signaling pathway, and JAK-STAT signaling pathway. It has been previously reported that inflammation can induce immune cell recruitment followed by activation of the JAK/STAT pathway, the primary signal transduction cascade utilized by cytokines essential for initiating innate immunity (38). Examples of cytokines that utilize the JAK-STAT pathway include erythropoietin, growth hormone, IL-2, IL-6, IL-7, and IFN, among other related molecules (39, 40). Interestingly, these findings were consistent with the results of a recent study of ASS Endotype 2, a DM-like cluster (41). For example, the expression levels of the *IFITM3* and *IFI6* genes were found to be the highest in patients with MDA5^+^ DM, followed by those with ASS, according to our scRNA-seq analysis (**Supplementary Figure 6D**). Our study revealed the shared pathogenic mechanisms of ASS disease and MDA5^+A^ DM, opening new avenues for research on IIM.

However, it is important to acknowledge certain limitations within our study. First, our sample size is relatively small; therefore, further validation through larger sample sizes is necessary. Second, some of the changes in gene expression and pathways observed in T cells may be attributable to the skewing of T cell populations present in each patient group (as shown in **Figures 2A–D**), rather than to disease group as a variable. Furthermore, the lack of samples from lesion sites for detailed analysis hinders a comprehensive understanding of the disease development mechanism. And *in vitro* or *in vivo* experiments are needed to elucidate the physiological functions of these cells and their associated molecular pathways more comprehensively.

Overall, our study provides valuable insights into the transcriptional profile of PBMCs in ASS. We utilized MDA5^+^ DM for disease control to investigate the similarities and differences between the two diseases. Through this investigation, we identified potential correlations between changes in specific cell proportions and ASS disease. These alterations in intercellular communication, along with differentially expressed genes, offer novel therapeutic targets that can contribute to the search for effective treatment regimens.

## Data Availability

The raw sequencing data from this study have been deposited in the Genome Sequence Archive in BIG Data Center (https://bigd.big.ac.cn/), Beijing Institute of Genomics (BIG), Chinese Academy of Sciences, under the accession number: PRJCA024528. Any additional information required to reanalyze the data reported in this paper is available from the lead contact upon request.

## Supplementary material

Supplementary Figure 1 Related to **Figure 1** **(A)** The top three genes in 13 different cell types. **(B)** Dot plot showing metabolic activation in patients with ASS and controls. **(C)** Pathway scores for both the ASS patient and control groups. ****p<0.0001; Wilcoxon rank sum test. **(D)** Volcano plot illustrating differentially expressed genes (DEGs) in each cell type between ASS patients and HCs through single-cell sequencing. Red dots represent genes with p-values <0.01, black dots represent genes with p-values >0.01, and blue fonts are genes of interest with p-values <0.01. **(E)** Volcano plot displaying DEGs in each cell type between ASS patients and MDA5^+^ DM patients according to single-cell sequencing analysis.

Supplementary Figure 2 Related to **Figure 2** **(A)** UMAP plot illustrating the normalized expression levels of the *CD4*, *CD8A*, and *CD8B* genes. **(B)** UMAP plot showing 19 groups reclustered from T cells and the pie chart showing the distribution of T-cell clusters. **(C)** Volcano plot displaying differentially expressed genes (DEGs) in MAIT cells between ASS patients and HCs. **(D)** The scores of the inflammatory response pathway, type I MHC pathway, and oxidative phosphorylation pathway in both the ASS patient group and the control group; ***p<0.001; Wilcoxon rank sum test. **(E)** Circos plots illustrating the intercellular communication network among T-cell subtypes in ASS patients and controls.

Supplementary Figure 3 Related to **Figure 3** **(A)** UMAP showing the subclusters of CD14^+^ monocytes and the distribution of CD14^+^ monocytes in patients with ASS and controls. **(B)** Bubble plots showing the expression patterns of markers in different CD14^+^ Mono cell types. **(C)** Bar charts displaying the relative abundance of CD14^+^ monocytes across different groups. **(D)** Volcano plot depicting the differentially expressed genes (DEGs) in CD14_Activated cells between ASS patients and HCs. **(E)** Bubble plots demonstrating the enrichment of GO pathways for CD14^+^ Mono cell subtypes. **(F)** UMAP plot revealing four distinct subtypes of neutrophils and their distribution in both ASS patients and controls. **(G)** UMAP plot illustrating the expression patterns of the markers associated with different neutrophil subtypes. **(H)** Bar charts showing the cluster abundance of neutrophils across different groups. **(J)** Volcano plot displaying DEGs in CAMP_Neu cells between ASS patients and HCs and GO enrichment analysis of CAMP_Neu cells from ASS patients. **(K)** UMAP showing developmental differentiation trajectories of neutrophils among these three groups.

Supplementary Figure 4 Related to **Figure 3** **(A)** Circos plots illustrating the CCL cell signaling networks between major cell types in patients with ASS and controls. **(B)** Determination of the maximum contribution of efferent signals from 13 cell populations. **(C)** Bubble plots displaying the differential target gene expression of CD14^+^ monocytes as receptors. **(D)** Bubble plot showing the differential target gene expression of proliferative NKT cells as the receptor.

Supplementary Figure 5 Related to **Figure 5** **(A)** Heatmap illustrating the heightened activity of transcription factors in T cells among patients with ASS and healthy controls. The symbol (+) denotes positive regulation, while (-) indicates negative regulation. **(B)** Heatmap depicting the enhanced activity of transcription factors in T cells from MDA5^+^ DM patients and healthy controls. The symbol (+) represents positive regulation, whereas (-) signifies negative regulation. **(C)** Violin plot displaying the expression level of IRF1 in ASS patients and controls; ****p<0.0001; ns, not statistically significant; Wilcoxon rank sum test. **(D)** UMAP plot showing the normalized expression of IRF1(+) across the three groups. **(E)** Violin plot showing the expression level of FOSB in ASS patients and controls; ****p<0.0001; **p<0.01; Wilcoxon rank sum test. **(F)** UMAP plot displaying the normalized expression of FOSB(+) across the three groups.

Supplementary Figure 6 Related to **Figures 6**, **7** **(A)** Distribution of the length of complementarity determining region 3 (CDR3) in patients with ASS, HCs, and MDA5^+^ DM disease controls. **(B)** Venn diagram illustrating the upregulated genes specific to the disease group. **(C)** UMAP plot displaying the normalized expression levels of the *CTSA* gene and *SOCS3* gene across the three groups. **(D)** Violin plot displaying the expression levels of the *IFITM3* gene and *IFI6* gene in ASS patients and controls.

## References

[1] Lundberg IE Fujimoto M Vencovsky J Aggarwal R Holmqvist M Christopher-Stine L. Idiopathic inflammatory myopathies. Nat Rev Dis Primers. (2021) 7(1):86. doi: 10.1038/s41572-021-00321-x 34857798

[2] McHugh NJ Tansley SL. Autoantibodies in myositis. Nat Rev Rheumatol. (2018) 14:290–302. doi: 10.1038/nrrheum.2018.56 29674612

[3] Wu W Collins BF Gardner GC Hippe DS Ho LA Raghu G. Antisynthetase syndrome-related interstitial lung disease (ASyS-ILD): longitudinal imaging findings. Eur Radiol. (2023) 33(7):4746–57. doi: 10.1007/s00330-023-09439-w 36786906

[4] Bauhammer J Fiehn C. Antisynthetase syndromes. Z Rheumatol. (2019) 78:645–55. doi: 10.1007/s00393-019-0665-0 31346706

[5] Gupta R Kumar S Gow P Hsien-Cheng Chang L Yen L. Anti-MDA5-associated dermatomyositis. Intern Med J. (2020) 50:484–7. doi: 10.1111/imj.14789 32270621

[6] Lu X Peng Q Wang G. Anti-MDA5 antibody-positive dermatomyositis: pathogenesis and clinical progress. Nat Rev Rheumatol. (2024) 20:48–62. doi: 10.1038/s41584-023-01054-9 38057474

[7] Castro-Molina SA Méndez-Flores S. Anti-MDA5 dermatomyositis. Literature review. Rev Med Inst Mex Seguro Soc. (2023) 61:99–105. 36542793 PMC10395958

[8] Chen F Wang D Shu X Nakashima R Wang G. Anti-MDA5 antibody is associated with A/SIP and decreased T cells in peripheral blood and predicts poor prognosis of ILD in Chinese patients with dermatomyositis. Rheumatol Int. (2012) 32:3909–15. doi: 10.1007/s00296-011-2323-y 22198664

[9] Stubbington MJT Rozenblatt-Rosen O Regev A Teichmann SA. Single-cell transcriptomics to explore the immune system in health and disease. Science. (2017) 358:58–63. doi: 10.1126/science.aan6828 28983043 PMC5654495

[10] Zhu L Cao Z Wang S Zhang C Fang L Ren Y. Single-cell transcriptomics reveals peripheral immune responses in anti-synthetase syndrome-associated interstitial lung disease. Front Immunol. (2022) 13:804034. doi: 10.3389/fimmu.2022.804034 35250976 PMC8891123

[11] Solomon J Swigris JJ Brown KK. Myositis-related interstitial lung disease and antisynthetase syndrome. Jornal brasileiro pneumologia: publicacao oficial da Sociedade Bras Pneumologia e Tisilogia. (2011) 37:100–9. doi: 10.1590/s1806-37132011000100015 PMC3676869 21390438

[12] Bohan A Peter JB. Polymyositis and dermatomyositis (second of two parts). New Engl J Med. (1975) 292:403–7. doi: 10.1056/nejm197502202920807 1089199

[13] Slovin S Carissimo A Panariello F Grimaldi A Bouché V Gambardella G. Single-cell RNA sequencing analysis: A step-by-step overview. Methods Mol Biol. (2021) 2284:343–65. doi: 10.1007/978-1-0716-1307-8_19 33835452

[14] Kumar N Mishra B Athar M Mukhtar S. Inference of gene regulatory network from single-cell transcriptomic data using pySCENIC. Methods Mol Biol. (2021) 2328:171–82. doi: 10.1007/978-1-0716-1534-8_10 34251625

[15] Van de Sande B Flerin C Davie K De Waegeneer M Hulselmans G Aibar S. A scalable SCENIC workflow for single-cell gene regulatory network analysis. Nat Protoc. (2020) 15:2247–76. doi: 10.1038/s41596-020-0336-2 32561888

[16] Schmitt P Sorin B Frouté T Parisot N Calevro F Peignier S. GReNaDIne: A data-driven python library to infer gene regulatory networks from gene expression data. Genes (Basel). (2023) 14:269. doi: 10.3390/genes14020269 36833196 PMC9957546

[17] Jin S Guerrero-Juarez CF Zhang L Chang I Ramos R Kuan CH. Inference and analysis of cell-cell communication using CellChat. Nat Commun. (2021) 12:1088. doi: 10.1038/s41467-021-21246-9 33597522 PMC7889871

[18] Browaeys R Saelens W Saeys Y. NicheNet: modeling intercellular communication by linking ligands to target genes. Nat Methods. (2020) 17:159–62. doi: 10.1038/s41592-019-0667-5 31819264

[19] Liu Z Sun D Wang C. Evaluation of cell-cell interaction methods by integrating single-cell RNA sequencing data with spatial information. Genome Biol. (2022) 23:218. doi: 10.1186/s13059-022-02783-y 36253792 PMC9575221

[20] Miao YR Zhang Q Lei Q Luo M Xie GY Wang H. ImmuCellAI: A unique method for comprehensive T-cell subsets abundance prediction and its application in cancer immunotherapy. Adv Sci (Weinh). (2020) 7:1902880. doi: 10.1002/advs.201902880 32274301 PMC7141005

[21] Wu Y Yang S Ma J Chen Z Song G Rao D. Spatiotemporal immune landscape of colorectal cancer liver metastasis at single-cell level. Cancer Discovery. (2022) 12:134–53. doi: 10.1158/2159-8290.Cd-21-0316 34417225

[22] Song L Cohen D Ouyang Z Cao Y Hu X Liu XS. TRUST4: immune repertoire reconstruction from bulk and single-cell RNA-seq data. Nat Methods. (2021) 18:627–30. doi: 10.1038/s41592-021-01142-2 PMC9328942 33986545

[23] Zhang F Li X Tian W. Unsupervised inference of developmental directions for single cells using VECTOR. Cell Rep. (2020) 32:108069. doi: 10.1016/j.celrep.2020.108069 32846127

[24] Hwang N Huh Y Bu S Seo KJ Kwon SH Kim JW. Single-cell sequencing of PBMC characterizes the altered transcriptomic landscape of classical monocytes in BNT162b2-induced myocarditis. Front Immunol. (2022) 13:979188. doi: 10.3389/fimmu.2022.979188 36225942 PMC9549039

[25] Hum RM Lilleker JB Lamb JA Oldroyd AGS Wang G Wedderburn LR. Comparison of clinical features between patients with anti-synthetase syndrome and dermatomyositis: Results from the MYONET registry. Rheumatol (Oxford). (2023) 63(8):2093–2100. doi: 10.1093/rheumatology/kead481 PMC11292049 37698987

[26] Legoux F Salou M Lantz O. MAIT cell development and functions: the microbial connection. Immunity. (2020) 53:710–23. doi: 10.1016/j.immuni.2020.09.009 33053329

[27] Zuo Y Ye L Chen F Shen Y Lu X Wang G. Different multivariable risk factors for rapid progressive interstitial lung disease in anti-MDA5 positive dermatomyositis and anti-synthetase syndrome. Front Immunol. (2022) 13:845988. doi: 10.3389/fimmu.2022.845988 35320936 PMC8936070

[28] Aouizerate J De Antonio M Bassez G Gherardi RK Berenbaum F Guillevin L. Myofiber HLA-DR expression is a distinctive biomarker for antisynthetase-associated myopathy. Acta Neuropathol Commun. (2014) 2:154. doi: 10.1186/s40478-014-0154-2 25339355 PMC4210467

[29] Zhao Q Hu Q Meng S Zhang Q Wang T Liu C. Metabolic profiling of patients with different idiopathic inflammatory myopathy subtypes reveals potential biomarkers in plasma. Clin Exp Med. (2023) 23:3417–29. doi: 10.1007/s10238-023-01073-6 PMC10618316 37103652

[30] Ye Y Chen Z Jiang S Jia F Li T Lu X. Single-cell profiling reveals distinct adaptive immune hallmarks in MDA5+ dermatomyositis with therapeutic implications. Nat Commun. (2022) 13:6458. doi: 10.1038/s41467-022-34145-4 36309526 PMC9617246

[31] Chávez MD Tse HM. Targeting mitochondrial-derived reactive oxygen species in T cell-mediated autoimmune diseases. Front Immunol. (2021) 12:703972. doi: 10.3389/fimmu.2021.703972 34276700 PMC8281042

[32] Jang DI Lee AH Shin HY Song HR Park JH Kang TB. The role of tumor necrosis factor alpha (TNF-α) in autoimmune disease and current TNF-α Inhibitors in therapeutics. Int J Mol Sci. (2021) 22:2719. doi: 10.3390/ijms22052719 33800290 PMC7962638

[33] Ivashkiv LB. IFNγ: signalling, epigenetics and roles in immunity, metabolism, disease and cancer immunotherapy. Nat Rev Immunol. (2018) 18:545–58. doi: 10.1038/s41577-018-0029-z PMC6340644 29921905

[34] Schroder K Hertzog PJ Ravasi T Hume DA. Interferon-gamma: an overview of signals, mechanisms and functions. J Leukoc Biol. (2004) 75:163–89. doi: 10.1189/jlb.0603252 14525967

[35] Rigolet M Hou C Baba Amer Y Aouizerate J Periou B Gherardi RK. Distinct interferon signatures stratify inflammatory and dysimmune myopathies. RMD Open. (2019) 5:e000811. doi: 10.1136/rmdopen-2018-000811 30886734 PMC6397431

[36] Pinal-Fernandez I Casal-Dominguez M Derfoul A Pak K Plotz P Miller FW. Identification of distinctive interferon gene signatures in different types of myositis. Neurology. (2019) 93:e1193–204. doi: 10.1212/wnl.0000000000008128 PMC6808530 31434690

[37] Galindo-Feria AS Albrecht I Fernandes-Cerqueira C Notarnicola A James EA Herrath J. Proinflammatory histidyl-transfer RNA synthetase-specific CD4+ T cells in the blood and lungs of patients with idiopathic inflammatory myopathies. Arthritis Rheumatol. (2020) 72:179–91. doi: 10.1002/art.41075 31403245

[38] O’Shea JJ Plenge R. JAK and STAT signaling molecules in immunoregulation and immune-mediated disease. Immunity. (2012) 36:542–50. doi: 10.1016/j.immuni.2012.03.014 PMC3499974 22520847

[39] O’Shea JJ Holland SM Staudt LM. JAKs and STATs in immunity, immunodeficiency, and cancer. N Engl J Med. (2013) 368:161–70. doi: 10.1056/NEJMra1202117 PMC7604876 23301733

[40] Leonard WJ O’Shea JJ. Jaks and STATs: biological implications. Annu Rev Immunol. (1998) 16:293–322. doi: 10.1146/annurev.immunol.16.1.293 9597132

[41] Wu S Xiao X Zhang Y Zhang X Zhang X Wang G Peng Q. Novel endotypes of antisynthetase syndrome identified independent of anti-aminoacyl transfer RNA synthetase antibody specificity that improve prognostic stratification. Ann Rheum Dis. (2024) 83(6):775–786. doi: 10.1136/ard-2023-225284 38395605

