# Supplementary figures and images for: A distinct immune landscape in anti-synthetase syndrome profiled by a single-cell genomic study
Source: Front Immunol. 2024 Oct 24;15:1436114. doi: 10.3389/fimmu.2024.1436114 (PMC11540782; doi:10.3389/fimmu.2024.1436114)

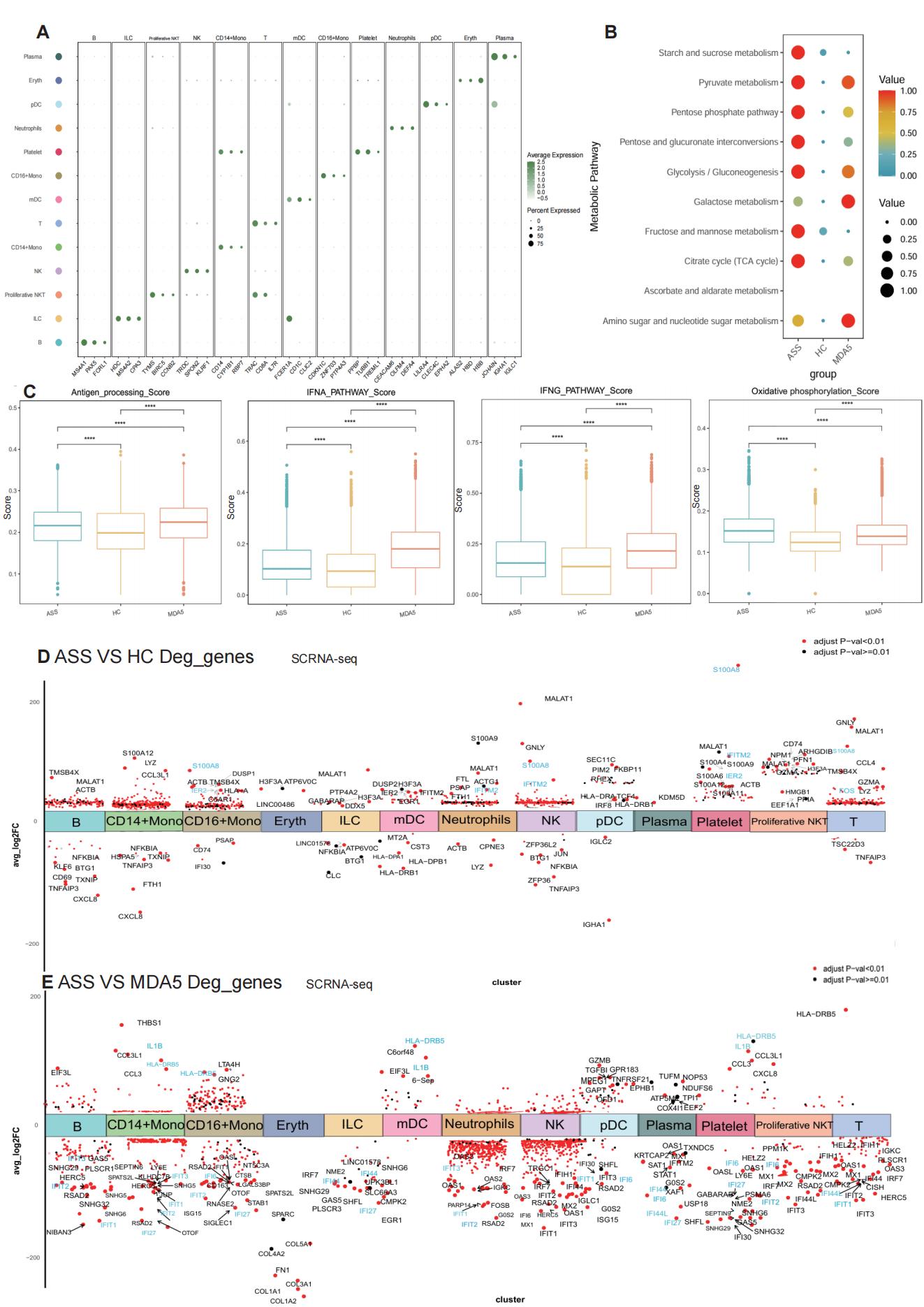

Supplement: Supplementary Figure 1 — Related to Figure 1 (A) The top three genes in 13 different cell types. (B) Dot plot showing metabolic activation in patients with ASS and controls. (C) Pathway scores for both the ASS patient and control groups. ****p<0.0001; Wilcoxon rank sum test. (D) Volcano plot illustrating differentially expressed genes (DEGs) in each cell type between ASS patients and HCs through single-cell sequencing. Red dots represent genes with p-values <0.01, black dots represent genes with p-values >0.01, and blue fonts are genes of interest with p-values <0.01. (E) Volcano plot displaying DEGs in each cell type between ASS patients and MDA5+ DM patients according to single-cell sequencing analysis. [file Image1.jpeg]

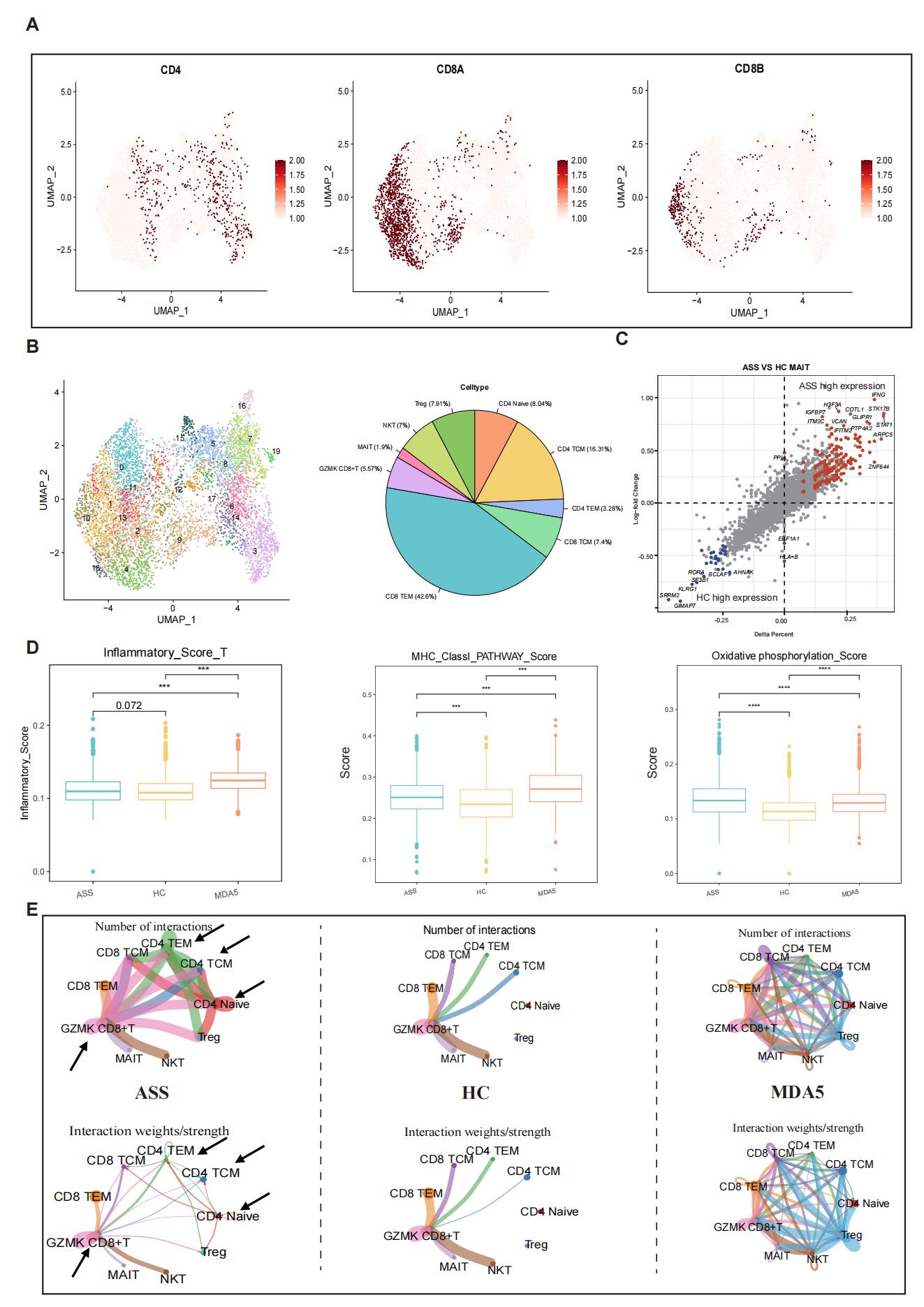

Supplement: Supplementary Figure 2 — Related to Figure 2 (A) UMAP plot illustrating the normalized expression levels of the CD4, CD8A, and CD8B genes. (B) UMAP plot showing 19 groups reclustered from T cells and the pie chart showing the distribution of T-cell clusters. (C) Volcano plot displaying differentially expressed genes (DEGs) in MAIT cells between ASS patients and HCs. (D) The scores of the inflammatory response pathway, type I MHC pathway, and oxidative phosphorylation pathway in both the ASS patient group and the control group; ***p<0.001; Wilcoxon rank sum test. (E) Circos plots illustrating the intercellular communication network among T-cell subtypes in ASS patients and controls. [file Image2.jpeg]

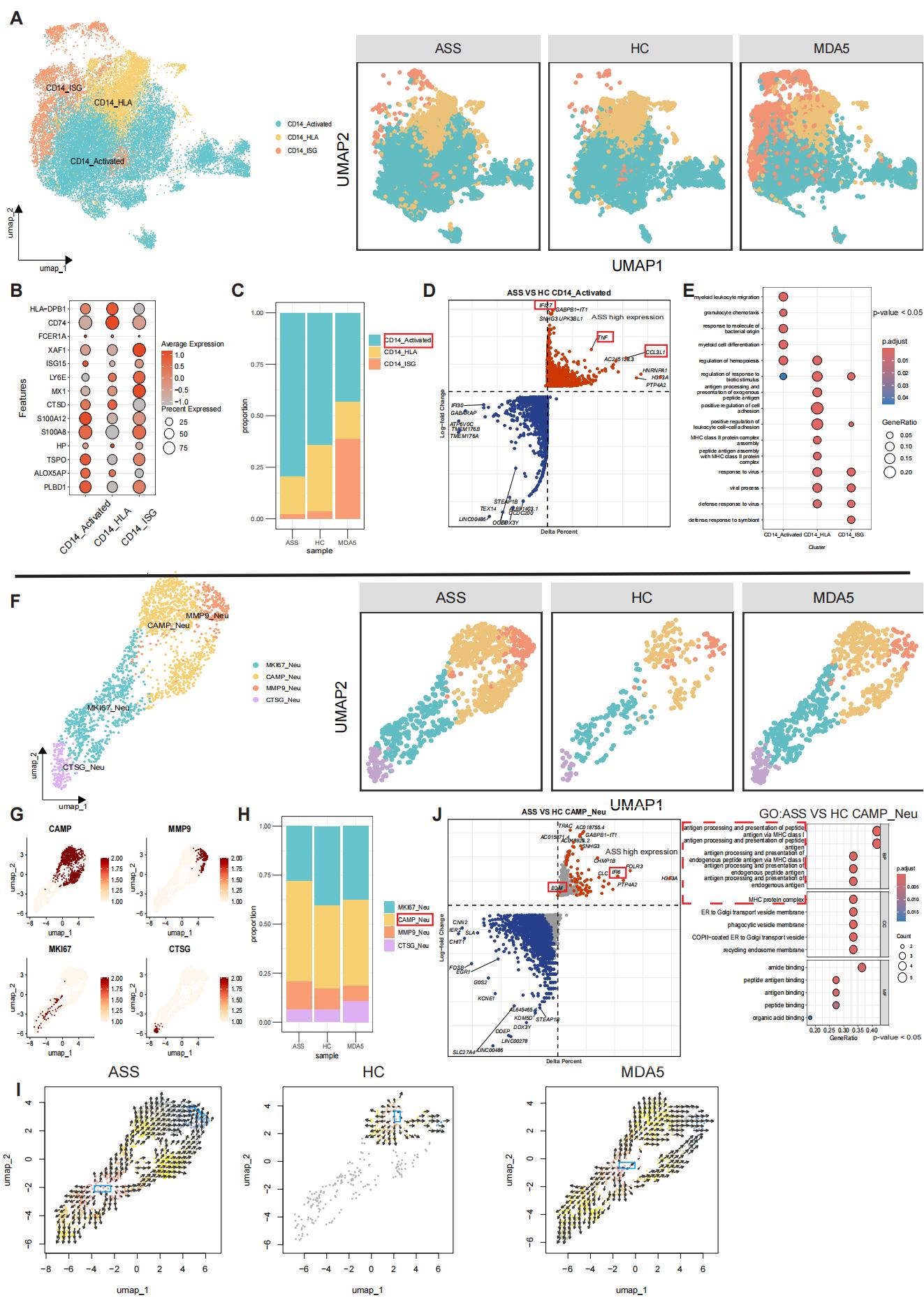

Supplement: Supplementary Figure 3 — Related to Figure 3 (A) UMAP showing the subclusters of CD14+ monocytes and the distribution of CD14+ monocytes in patients with ASS and controls. (B) Bubble plots showing the expression patterns of markers in different CD14+ Mono cell types. (C) Bar charts displaying the relative abundance of CD14+ monocytes across different groups. (D) Volcano plot depicting the differentially expressed genes (DEGs) in CD14_Activated cells between ASS patients and HCs. (E) Bubble plots demonstrating the enrichment of GO pathways for CD14+ Mono cell subtypes. (F) UMAP plot revealing four distinct subtypes of neutrophils and their distribution in both ASS patients and controls. (G) UMAP plot illustrating the expression patterns of the markers associated with different neutrophil subtypes. (H) Bar charts showing the cluster abundance of neutrophils across different groups. (J) Volcano plot displaying DEGs in CAMP_Neu cells between ASS patients and HCs and GO enrichment analysis of CAMP_Neu cells from ASS patients. (K) UMAP showing developmental differentiation trajectories of neutrophils among these three groups. [file Image3.jpeg]

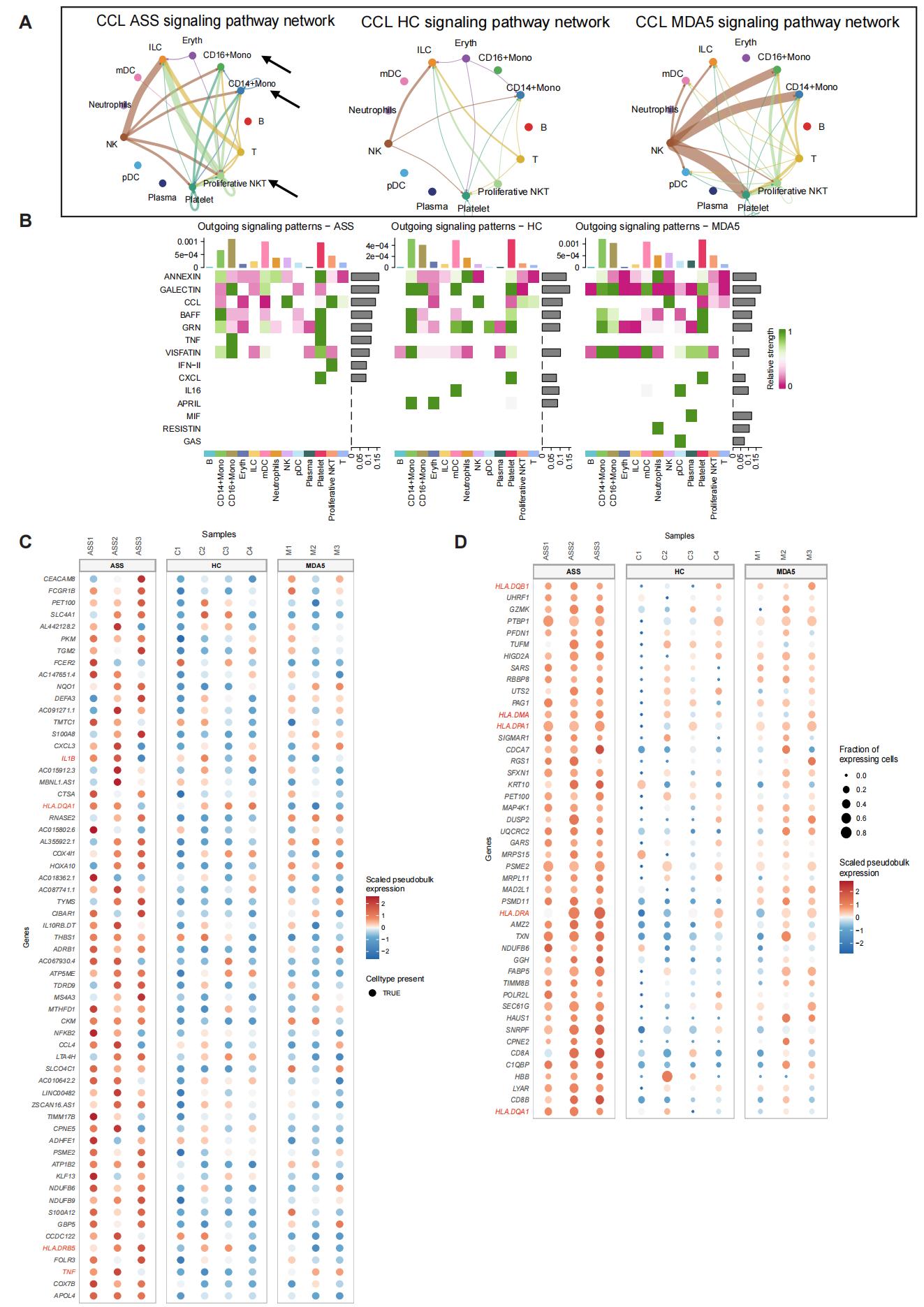

Supplement: Supplementary Figure 4 — Related to Figure 3 (A) Circos plots illustrating the CCL cell signaling networks between major cell types in patients with ASS and controls. (B) Determination of the maximum contribution of efferent signals from 13 cell populations. (C) Bubble plots displaying the differential target gene expression of CD14+ monocytes as receptors. (D) Bubble plot showing the differential target gene expression of proliferative NKT cells as the receptor. [file Image4.jpeg]

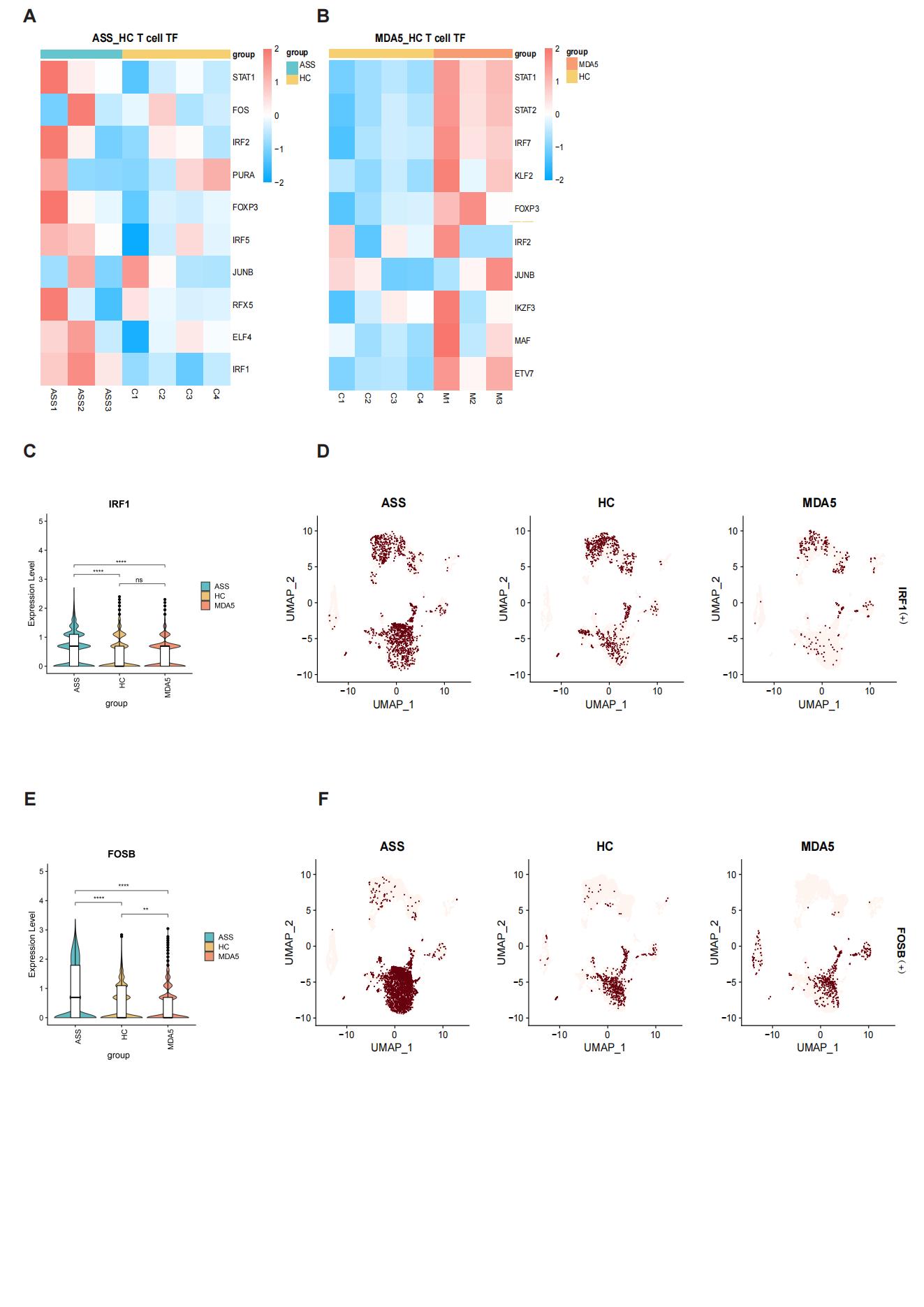

Supplement: Supplementary Figure 5 — Related to Figure 5 (A) Heatmap illustrating the heightened activity of transcription factors in T cells among patients with ASS and healthy controls. The symbol (+) denotes positive regulation, while (-) indicates negative regulation. (B) Heatmap depicting the enhanced activity of transcription factors in T cells from MDA5+ DM patients and healthy controls. The symbol (+) represents positive regulation, whereas (-) signifies negative regulation. (C) Violin plot displaying the expression level of IRF1 in ASS patients and controls; ****p<0.0001; ns, not statistically significant; Wilcoxon rank sum test. (D) UMAP plot showing the normalized expression of IRF1(+) across the three groups. (E) Violin plot showing the expression level of FOSB in ASS patients and controls; ****p<0.0001; **p<0.01; Wilcoxon rank sum test. (F) UMAP plot displaying the normalized expression of FOSB(+) across the three groups. [file Image5.jpeg]

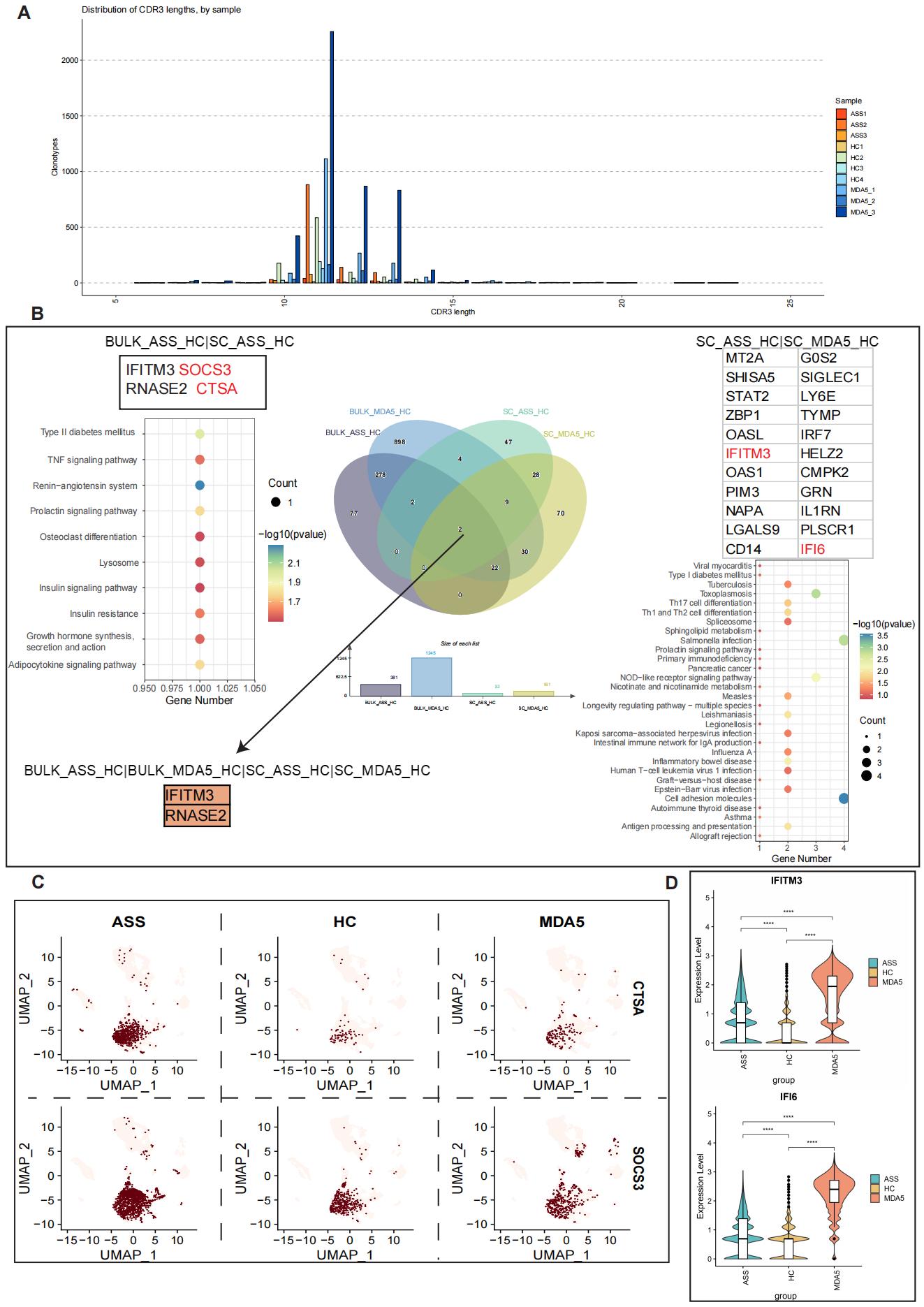

Supplement: Supplementary Figure 6 — Related to Figures 6 , 7 (A) Distribution of the length of complementarity determining region 3 (CDR3) in patients with ASS, HCs, and MDA5+ DM disease controls. (B) Venn diagram illustrating the upregulated genes specific to the disease group. (C) UMAP plot displaying the normalized expression levels of the CTSA gene and SOCS3 gene across the three groups. (D) Violin plot displaying the expression levels of the IFITM3 gene and IFI6 gene in ASS patients and controls. [file Image6.jpeg]
