# Supplementary material for: A distinct immune landscape in anti-synthetase syndrome profiled by a single-cell genomic study
Source: Front Immunol. 2024 Oct 24;15:1436114. doi: 10.3389/fimmu.2024.1436114 (PMC11540782; doi:10.3389/fimmu.2024.1436114)
Supplement: Supplementary file 7 [file Table1.docx]

**Supplementary Table 1**

***Clinical information of the recruited donors for scRNA-seq***

| Sample ID | Sex/Age (year) | Disease Duration (month) | MSA | CK(U/L) | CRP (mg/dl) | ILD |
| --- | --- | --- | --- | --- | --- | --- |
| ASS1 | F/37 | 12 | Jo-1 | 721 | 0.25 | Yes |
| ASS2 | F/64 | 3 | Jo-1 | 393 | 1.07 | Yes |
| ASS3 | F/63 | 4 | Jo-1 | 6871 | 1.26 | Yes |
| MDA5_1 | M/58 | 2 | MDA5 | 306 | 1.57 | Yes |
| MDA5_2 | F/54 | 1 | MDA5 | 351 | 0.1 | Yes |
| MDA5_3 | F/58 | 0.5 | MDA5 | 466 | 0.62 | Yes |
| HC1 | M/43 | NA | NA | NA | NA | NA |
| HC2 | F/31 | NA | NA | NA | NA | NA |
| HC3  HC4 | M/67  F/61 | NA  NA | NA  NA | NA  NA | NA  NA | NA  NA |

Abbreviations: MSA, myositis-specific autoantibody; CK, creatine kinase; CRP, C reactive protein; ILD, interstitial lung disease.
