# Supplementary material for: A distinct immune landscape in anti-synthetase syndrome profiled by a single-cell genomic study
Source: Front Immunol. 2024 Oct 24;15:1436114. doi: 10.3389/fimmu.2024.1436114 (PMC11540782; doi:10.3389/fimmu.2024.1436114)
Supplement: Supplementary file 8 [file Table2.docx]

**Supplementary Table 2**

***Demographic data of recruited donors for MAIT cell validation (flow cytometry)***

|  | HCs (n=14) | ASS(n=12) |
| --- | --- | --- |
| Age, mean (range) | 54.64 (33-71) | 56.25 (35-79) |
| Sex (male/female) | 4/10 | 4/8 |
| Anti-Jo-1 | NA | 8 |
| Anti-EJ-1 | NA | 2 |
| Anti-PL-7 | NA | 1 |
| Anti-PL-12 | NA | 1 |
| CK (U/L), mean (range) | NA | 1152 (54-6871) |
| CRP mean (range) | NA | 2.25 (0.25-8.64) |
| ILD (%) | NA | 7/12 |

Abbreviations: CK, creatine kinase; CRP, C reactive protein; ILD, interstitial lung disease.
